# Capillary Skimming of Floating Microplastics via a Water‐Bridged Ratchet

**DOI:** 10.1002/advs.202408623

**Published:** 2024-11-05

**Authors:** Seohyun Cho, Sang Jin Park, Young Jin Lee, You Jun Lee, Young A Lee, Ho‐Young Kim, Seong Jin Kim, Seok Chung, Myoung‐Woon Moon

**Affiliations:** ^1^ Extreme Materials Research Center Korea Institute of Science and Technology (KIST) Seoul 02792 Republic of Korea; ^2^ School of Mechanical Engineering Korea University Seoul 02841 Republic of Korea; ^3^ Department of Mechanical Engineering Seoul National University Seoul 08826 Republic of Korea; ^4^ KU‐KIST Graduate School of Converging Science and Technology Korea University Seoul 02841 Republic of Korea; ^5^ Korea Institute of Science and Technology – Sungkyunkwan University Carbon‐Neutral Research Center Sungkyunkwan University (SKKU) Suwon 16419 Republic of Korea; ^6^ School of Chemical Engineering Sungkyunkwan University (SKKU) Suwon 16419 Republic of Korea

**Keywords:** capillary skimming, cheerios effect, microplastics removal, water‐bridged ratchet, water meniscus

## Abstract

Floating microplastics (MPs) have recently become a major concern in marine pollution; however, current filter‐based technology is hardly effective for directly removing such MPs from the water surface because of specific mesh size and clogging issues. This paper introduces a new skimming concept for removing floating MPs utilizing capillary force mediated by the elevation of a hydrophilic ratchet at the air−water interface. MPs floating near the ratchet surface are spontaneously forced toward the ratchet with a concave water meniscus, driven by the Cheerios effect. The MPs can then be skimmed and temporarily held by the deforming concave water meniscus as the ratchet rises. Here, it is found that the stability of the water bridge plays a crucial role in skimming success because it provides capillary adhesion between the MP and the ratchet. The proposed capillary skimming method is observed to be effective across nearly all types of floating MPs, ranging in size from 1 to 4 mm, and with densities varying from 0.02 to 0.97 g cm^−^^3^, which is also demonstrated by a prototype of marine robot cleaner.

## Graphical abstract

This study introduces a novel capillary skimming mechanism using a hydrophilic ratchet to remove floating microplastics (MPs) from water. The present mechanism uses capillary forces to attract and hold MPs, with converting the water meniscus into a water bridge while skimming. The skimming by the hydrophilic ratchet drum is found to be effective for MPs of various sizes and densities.

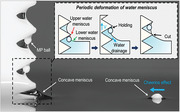

## 1 Introduction

Marine plastic pollution is a growing global environmental concern as a result of the exponential increase in plastic production.^[^ ^1^, ^2^, ^3^, ^4^ ^]^ The global annual output of plastics surpassed 300 million tons as of 2016.^[^ ^5^ ^]^ Plastic usage has increased 20‐fold over the last 50 years,^[^ ^4^ ^]^ which has unfortunately been accompanied by an increase in global plastic pollution caused by the uncontrolled release of tons of plastic waste not only on land but also in water.^[^ ^6^ ^]^ It was estimated in 2017 that 4.8 to 12.7 metric tons of plastic litter enters the ocean environment each year.^[^ ^7^, ^8^ ^]^ Marine plastic can be classified into five types by its size: mega‐plastics (>100 mm in size), macro‐plastics (>20 mm), meso‐plastics (5–20 mm), micro‐plastics (MPs; <5 mm) and nano‐plastics (<1 µm).^[^ ^7^, ^9^, ^10^ ^]^ Among them, MPs have received special attention due to their long degradation time, high mobility and small size. MPs are derived from two sources: preproduction plastics such as pellets or nurdles and weathered or mechanically degraded plastic materials.^[^ ^3^, ^4^, ^11^ ^]^ As MPs are small and have various shapes or colors, they are easily consumed by marine species, such as planktivores, fish, mussels, seabirds, and worms.^[^ ^1^, ^4^, ^6^, ^7^ ^]^ As humans are the top‐level predators in the food chain, such ingested MPs eventually end up in humans via food chain transmission.^[^ ^12^, ^13^, ^14^, ^15^ ^]^ Various types of MPs have been detected in foods and drinking water, as well as human stool.^[^ ^5^ ^]^ MPs have also been reported to absorb harmful substances such as heavy metals and concentrate pollutants in the environment.^[^ ^5^, ^16^, ^17^ ^]^ Furthermore, ingestion of MPs can directly cause blockages throughout the fish digestive system, resulting in structural and functional deterioration of the gastrointestinal tract.^[^ ^16^, ^18^, ^19^ ^]^

Thus far, the removal of such harmful MPs has been studied with the primary goal of developing a method to separate (or sample) MPs from other environmental matrices (soil, sediments, water, etc.) or biological materials.^[^ ^20^ ^]^ Density‐based separation such as flotation or elutriation, is a simple method for separating less dense MPs, such as polystyrene (PS), polyethylene (PE), and polypropylene (PP), from heavier materials, including denser MPs such as polyvinyl chloride (PVC) and nylon.^[^ ^20^, ^21^, ^22^, ^23^ ^]^ However, such density‐based separation is inaccurate in precisely separating each material because buoyancy can be altered in aquatic environments, due to surface fouling, bubble attachment, and hetero‐aggregation of MPs with organic matter.^[^ ^10^, ^18^, ^20^ ^]^ Size‐based separation, such as filtration or sieving, is the most commonly used method for separating MPs; however, it faces significant challenges such as clogging, particularly from biofilms in seawater.^[^ ^16^, ^18^, ^19^, ^24^ ^]^ In addition to the clogging issue that reduces filtration efficiency, dense membranes required to filter smaller MPs result in low flux due to their low permeability. Recent advances, such as high‐flux membrane systems, have shown promise in addressing these challenges by improving filtration rates and mitigating fouling effects.^[^ ^25^ ^]^ Electrochemical techniques such as electrocoagulation, which uses high‐frequency electricity to induce heat to coagulate MPs and then filter them, have also been used to separate MPs.^[^ ^4^ ^]^

Most of the current efforts have been dedicated to purifying MP‐containing water and reducing plastic waste through recycling; however, little attention has been given to recovery methods for directly removing MPs from the ocean. Filters called Neuston nets and Manta nets, with a mesh size of 200−505 µm, are used for the collection of floating MPs in the marine environment.^[^ ^26^, ^27^, ^28^ ^]^ However, such filtering methods are inevitably limited by the mesh size and cannot effectively achieve the simultaneous recovery of MPs with various sizes ranging from 1 µm to 5 mm. A smaller mesh size may help capture more MPs, but it is believed to cause a substantial increase in pressure, leading to mesh damage and increased susceptibility to bio‐fouling.^[^ ^27^, ^28^ ^]^ In addition to the filtration approach, a jellyfish‐inspired robotic grasper was used to directly collect MPs on site, employing a combination of electrohydraulic actuators and a hybrid structure consisting of both rigid and soft components.^[^ ^29^ ^]^

Pollutant recovery in the ocean has primarily been performed to remove liquid‐type pollutants of oil spilled on the water surface.^[^ ^30^ ^]^ Among automation devices, adhesion skimmers are widely used in practical oil spill treatment; these devices operate based on high adhesion between oil and the oleophilic solid surface of the skimmer.^[^ ^31^ ^]^ However, such an adhesion‐based principle is hardly applicable for skimming solid MPs on the water surface, as MPs are not expected to adhere to the surface. Recent oil‐recovery research also introduced the use of an oil scooper that filters water only through a hydrophilic filter.^[^ ^32^, ^33^, ^34^ ^]^ However, such scooping‐based water filtration becomes less effective for filtering MPs as the size of the plastic particles decreases.

**Figure 1:**
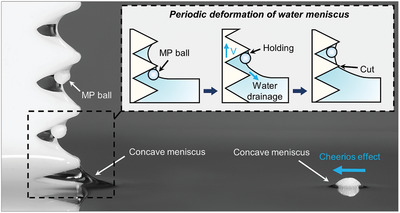
The skimming mechanism of a hydrophilic ratchet for an MP ball. The presence of nearby concave menisci induces a Cheerios effect, leading to the spontaneous attraction of an MP ball toward the ratchet. The schematic shows the subsequent skimming process of the MP ball with periodic deformation of the water meniscus. The skimmed MP balls are then firmly secured in the water‐bridged ratchet by capillary adhesion.

In this paper, we present a new mechanism of capillary skimming via a hydrophilic ratchet, which allows us to collect MPs floating at the water surface in an open environment (Movie S1, Supporting Information). Capillary skimming of floating MPs is enabled via a hydrophilic ratchet (**Figure** **1**). The nearby concave menisci formed by the ratchet structure induce a Cheerios effect, causing MPs located adjacent to the ratchet to be spontaneously attracted toward the ratchet. Afterward, as the ratchet rises, the MPs can be skimmed off and temporarily captured by a deforming concave water meniscus. The skimmed MPs are then stably capillary‐adhered to the water‐bridged ratchet. The size of the ratchet structure is designed to be on the order of the capillary length so that the shape of the water bridge formed in the ratchet structure can be easily controlled as the ratchet lifts out of or dips into the water. A transition from an asymmetric water meniscus to a water bridge surrounding the MPs must be accomplished to skim the MPs off the free surface of the water. For this, the ratchet structure is beneficial, in comparison to a flat plate, as it can induce inward motion of the asymmetric water meniscus inside the ratchet structure and cut it to prevent further gravitational water drainage. After the MPs are skimmed off, the negatively curved water bridge inside the ratchet provides secure capillary adhesion of the MPs to the ratchet, which is measured to be at least 230 times stronger than that on a flat plate. This secure capillary adhesion can later be released by dipping the ratchet back into water, which opens the capillary water bridge by allowing the flow of water into the ratchet (Movie S2, Supporting Information). The current mechanism, in addition to being applied to MP skimming, can be also used for density‐based MP separation by switching the floating medium from water to another liquid.

## 2 Results and Discussion

### 2.1 Capillary Skimming on a Flat Vertical Plate

**Figure 2:**
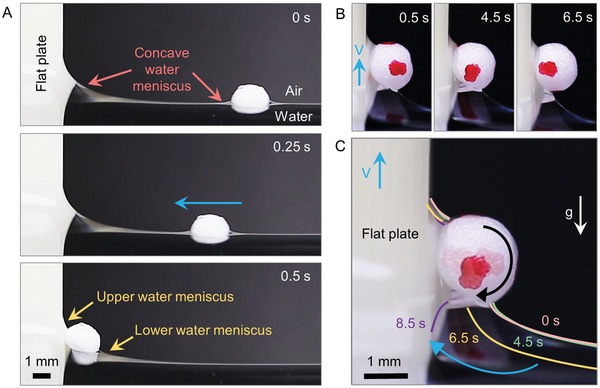
The skimming mechanism of a hydrophilic flat plate for an MP ball. (A) The sequential images show the approach of an EPS foam ball toward a flat plate between 0 and 0.5 seconds. Note that when the ball makes contact with the plate surface, the water meniscus becomes asymmetrical, consisting of an upper water bridge and a lower water meniscus. (B) The sequential images present the temporal evolution of the upper water bridge and lower water meniscus around the ball as the flat plate moves upward after the ball has touched the plate surface. (C) Composite image of the experimental pictures in (B).

We begin by examining the capillary skimming of floating MP of expanded polystyrene (EPS) foam ball by a vertically situated, flat plate (**Figure** **2**A). The plate surface had strong hydrophilicity with a zero equilibrium contact angle (CA) with water (Figure S1, Supporting Information), which was imparted by the oxygen plasma treatment (see the Experimental section). As a 2‐mm EPS foam ball had a much lower density (0.02–0.04 g cm^−3^) than water (≈1 g cm^−3^), it was surrounded by a concave water meniscus, as shown at 0 s in Figure 2A. This concave water meniscus induced an attraction force between the EPS foam ball and the plate, which had a similar concave water meniscus; this mechanism is commonly referred to as the Cheerios effect.^[^ ^35^, ^36^ ^]^ When these two concave water menisci overlapped, the ball began to accelerate toward the plate (Figure S2, Supporting Information). The 2‐mm EPS foam ball traveled at a maximum speed of 54 mm s^−1^ with an average speed of 11 mm s^−1^ over a distance of 9.3 mm in 0.5 s (Figure 2A). Upon impact with the plate, the ball was surrounded by an asymmetric water meniscus. Please note that in this study, the water bridge between the plate and the ball is referred to as the “upper water bridge,” while the water meniscus in contact with the free surface of water is referred to as the “lower water meniscus”.

As the plate lifted at a speed of 1 mm s^−1^, it resulted in a slight lifting of the ball (Figure 2B). Here, it is important to point out that the behaviors of the upper water bridge and lower water meniscus surrounding the ball differed from each other. The lower water meniscus traveled a longer distance in a clockwise direction (as shown in Figure 2C and Movie S3, Supporting Information), compared to the relatively stationary upper water bridge. The traces of the water menisci are marked by the magenta (0 s), green (4.5 s), orange (6.5 s), and purple curves (8.5 s). Here, the change in the shape of the lower water meniscus was attributed to gravitational drainage of water as the ball was lifted, which also led to a receding of the water contact line along the bottom portion of the ball. At the end, the water meniscus was converted into a water bridge connecting the ball and the plate (as shown by the purple curve at 8.5 s).

### 2.2 Holding and Skimming

**Figure 3:**
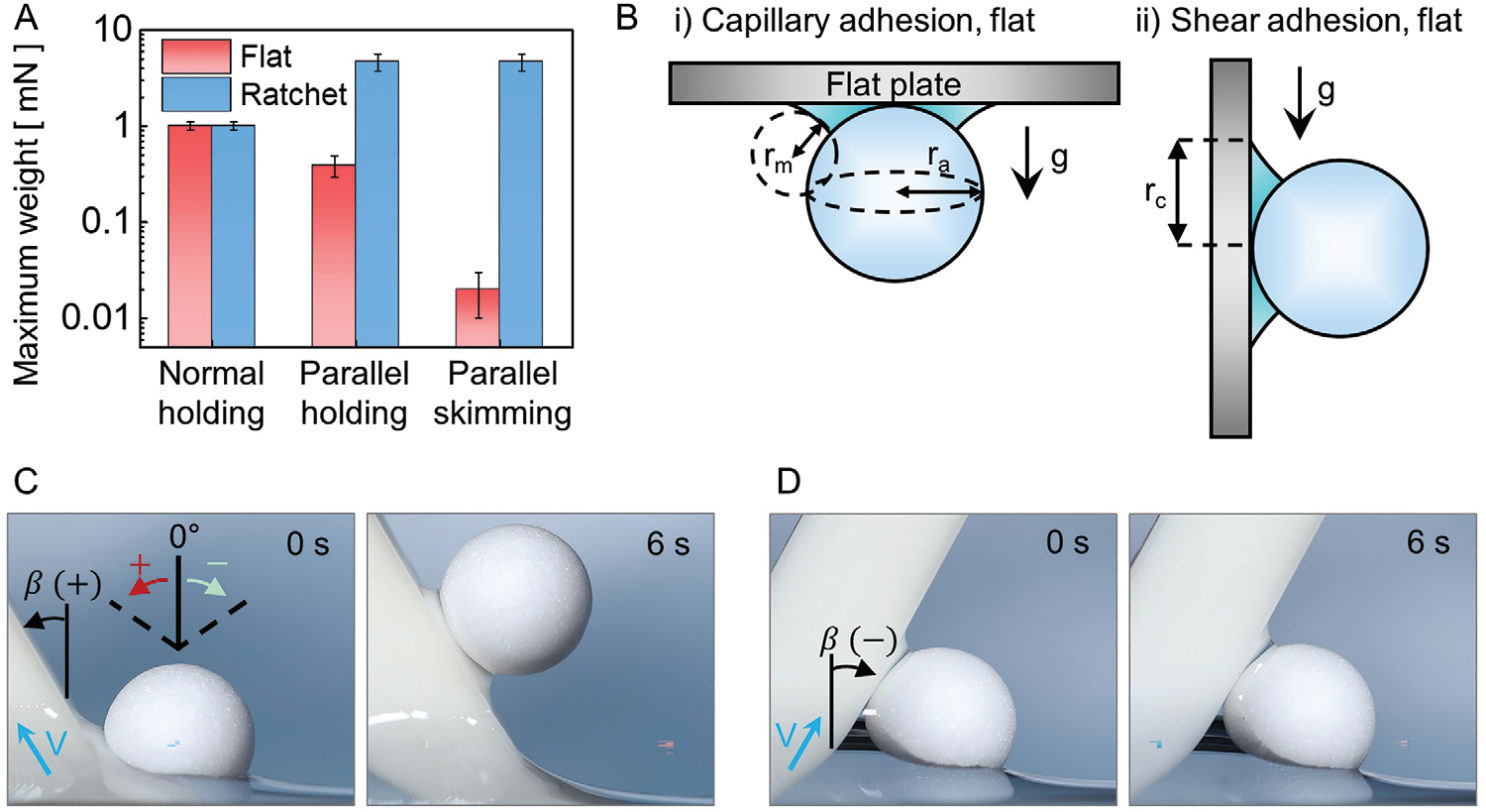
Capillary adhesion and shear adhesion between an MP ball and a plate surface. (A) A bar graph of the maximum weight that can be held or skimmed by a flat or ratchet plate under two different gravitational directions. (B) Schematics of (i) capillary and (ii) shear adhesion, where gravity acts normal and parallel to the plate surface respectively. (C,D) Skimming experiments with positive and negative tilting angles (β); (C) A 4‐mm EPS foam ball is successfully skimmed by lifting the plate with a positively tilted angle of + 30° (D), while it fails to skim at a negatively tilted angle of − 30°.

The maximum weight of an MP ball that a plate can support against gravity was measured under different geometric configurations (**Figure** **3**A). In the figure, “holding” indicates a test conducted by manually placing an MP ball on the flat plate, with a water bridge already between the components, and observing whether the ball fell due to gravity (Figure 3B, i,ii; Figure S3, Supporting Information). In contrast, “skimming” refers to a test where the plate is lifted to skim a ball from the free surface of the water (see Figure 2B,C). The terms “parallel” and “normal” refer to the direction of gravity relative to the plate. In this regard, the “normal‐holding” data (Figure 3A) refer to the maximum weight (1.0 ± 0.1 mN) that a plate can hold against gravity acting normal to the plate (Figure 3B, i). However, when gravity acted parallel to the plate surface (Figure 3B, ii), the maximally supportable weight drastically decreased to 0.4 ± 0.1 mN (parallel‐holding data in Figure 3A). This observation provides clear evidence that there were distinct behaviors in normal and shear adhesion by the capillary water bridge. This is because sliding can occur without breaking the water bridge. Rather, sliding can be initiated as long as the contact angle is greater than the advancing contact angle of *θ*_a_, which requires slight deformation of the water bridge. The difference between normal‐holding and parallel‐holding will be elaborated upon further in the theoretical analysis section below.

### 2.3 Effect of Lifting Angle on Capillary Skimming

Further comparison of the holding and skimming data on the flat plate (Figure 3A) clearly shows that a smaller critical mass was required to skim the ball (parallel‐skimming) than to hold it (parallel‐holding). Here, the main experimental difference was that the ball was placed on the free surface of the water at the beginning of the skimming experiment, while it was already positioned on the plate for the holding experiment. In the skimming experiment, initially, the ball was encircled by an asymmetric water meniscus (0.5 s in Figure 2A). Since after the ball was skimmed, capillary skimming did not occur before the water bridge formed, this implied that a rapid transition from the asymmetric water meniscus to the water bridge would make it easier to skim the ball off the free surface of water. When the plate was lifted with a tilting angle *β* in the + direction (Figure 3C) rather than the – direction (Figure 3D), a smaller amount of water needed to be drained from the lower water meniscus for the transition from the asymmetric meniscus to the water bridge. Furthermore, an increased positive tilting angle of *β* led to easier removal of the ball from the free surface of the water. A 4‐mm EPS foam ball could be skimmed when *β* was + 30° (Figure 3C), while MP skimming failed when *β* was − 30° (Figure 3D) under a lifting speed (V) of 10 mm s^−1^.

### 2.4 Capillary Skimming via Ratchet

**Figure 4:**
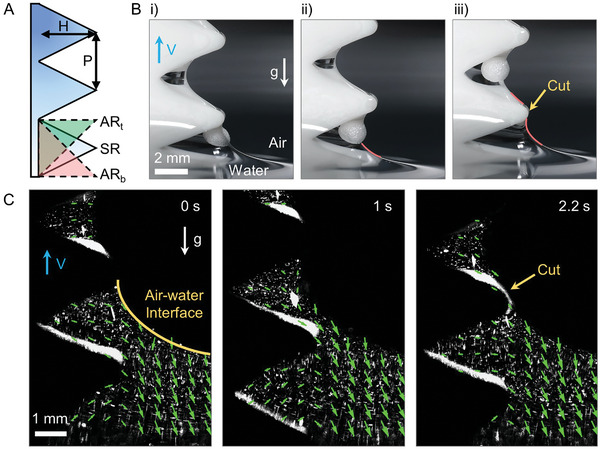
Capillary skimming via hydrophilic ratchet. (A) Schematic of the ratchet geometry with a pitch distance of P (peak‐to‐peak distance) and a peak height of H. Three different types of ratchets are presented: an asymmetric ratchet with the tip at the top (ARt), a symmetric ratchet with the tip in the middle (SR), and an asymmetric ratchet with the tip at the bottom (ARb). (B) Sequential images presenting the capillary skimming mechanism by which a ratchet‐structured plate with P = H = 6 mm skims a 2‐mm EPS foam ball. The yellow arrow indicates that the water meniscus is cut at the ratchet tip, which helps inhibit further drainage of water. (C) This effect is more clearly shown by visualizing the flow field of water that has settled inside the ratchet after the ratchet tip cutting effect.

Based on the observations presented in the previous section, it can be inferred that lifting the plate at a positive tilting angle is favorable for capillary skimming compared to lifting it at a negative angle. However, a 3‐mm polypropylene (PP) ball (0.9 g cm^−3^) failed to be skimmed even at a favorable lifting angle of + 60° (Figure S4, Supporting Information). To further enhance the skimming capability, we proposed utilizing a ratchet structure that concurrently employs both positive and negative tilting angles. The utilization of a ratchet structure enabled the capillary skimming of a 3‐mm PP ball, which had previously failed using a flat plate (Figure S5, Supporting Information). The schematic shows a unit ratchet configuration with a pitch (P, peak‐to‐peak distance) of a few millimeters (**Figure** **4**A), where H denotes the height of the peak. The wettability of the ratchet surface was identical to that on the flat plate, which was hydrophilic (Figure S6, Supporting Information). However, unlike the flat plate on which a thin water film was formed, the ratchet structure had a larger water pocket inside, known as a capillary water bridge (Figure S5, Supporting Information).^[^ ^37^ ^]^ The EPS foam ball was attracted toward the ratchet due to the Cheerios effect and subsequently pulled inside the ratchet (Movie S2, Supporting Information). As the ratchet was lifted up, water drained at the lower water meniscus, causing it to recede until the next ratchet tooth reached the free surface of the water (Figure 4B). After that, the lower water meniscus was *cut*, resulting in the formation of the water bridge (Figure 4B, iii), magenta contour). Water drainage stopped after the water meniscus was *cut* by the ratchet tip (orange arrow in Figure 4C), as shown by the disappearance of velocity vectors inside the ratchet, which was obtained by the particle image velocimetry (PIV) experiment (Figure 4C and Movie S4, Supporting Information). Such meniscus cutting was not available on a flat surface without a ratchet structure. As a result, when the flat plate was lifted up, the water on its surface continued to drain (Movie S5, Supporting Information). The meniscus cutting was also observed to help skim agglomerated MPs in various sizes and shapes by preventing the upper meniscus from being overloaded with too many MPs as shown in Figure S7 (Supporting Information).

EPS foam balls with a diameter of 4–6 mm were skimmed using four different types of plates: i) a hydrophobic flat plate, ii) a hydrophobic ratchet plate, iii) a hydrophilic flat plate, and iv) a hydrophilic ratchet plate (Figure S8, Supporting Information). It was further observed that the hydrophobic flat plate i) was incapable of skimming even a single MP ball, but the hydrophilic flat plate ii) skimmed a small number of balls owing to capillary adhesion. In contrast, both ratchet‐structured plates, regardless of wettability, skimmed a significantly larger number of balls. The skimming results for three sets of MP balls with different diameters (1.5–2.0, 3.0–5.0, and 6.0–8.0 mm) also showed that both ratchet plates were effective for skimming (Figure S8, Supporting Information). Therefore, it can be concluded that the ratchet structure plays a far more important role in effectively skimming MPs than does the hydrophilicity of the surface.

**Figure 5:**
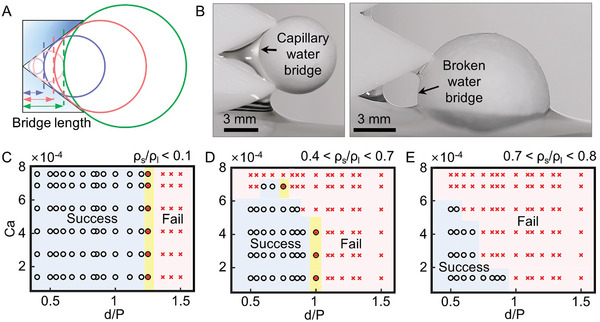
Role of capillary water bridge in skimming capability. (A) The schematic shows that the distance between the surface of the ball and the innermost point of the ratchet (i.e., the bridge length) increases as the ball diameter increases for a given ratchet geometry. (B) The optical images show a stable water bridge (left) and a broken bridge (right) when the ball diameter exceeds a critical threshold, resulting in skimming failure. (C–E) The regime maps present the success/failure of skimming with respect to the ratio of ball diameter to ratchet pitch, denoted as d/P (where P = H), and the capillary number (Ca = µV/σ) for different ratios of fluid density to ball density. Skimming success is denoted by the symbol “⚬” in the regimes, while skimming failure is represented by the symbol “x.”.

**Figure 6:**
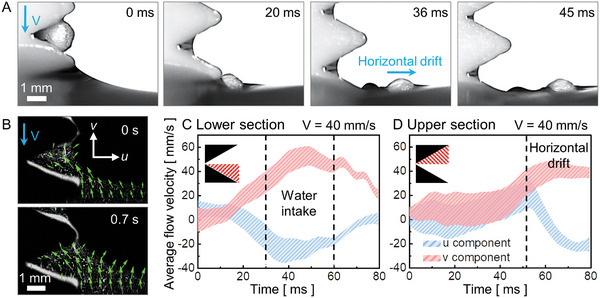
Release of capillary adhesion by intaking water into the ratchet. (A) Sequential images present a 2‐mm EPS foam ball captured by a water bridge in a ratchet tooth with P = H = 3 mm that is released with a horizontal drift away from the ratchet when it dips into the water. (B) The PIV experiment indicates that a water flow is generated in a direction to push the ball away from the ratchet as it submerges into the water. (C‐D) Temporal evolution of water flow velocity, which is averaged spatially for the (C) lower and (D) upper sections in a ratchet tooth, reveals that (C) water is introduced at a fast speed of ≈60 mm s^−1^ into the ratchet via the lower section (30–60 ms). (D) This water flow is later converted to push the ball away from the ratchet, with positive u and v components in the flow, as shown at ≈53 ms.

As the ball diameter increased for a given ratchet size, the *water bridge length*, which is defined as the distance from the innermost point of the ratchet to the surface of the ball also increased (**Figure** **5**A). This increase in water bridge length, especially when exceeding the capillary length (≈2.7 mm), is believed to deteriorate the stability of the capillary water bridge, causing it to break up.^[^ ^38^, ^39^ ^]^ The ratchet plate with a ratchet size of 6 mm failed to skim an EPS foam ball as the ball size increased from 7 mm (left) to 10 mm (right), which led to the break‐up of the inside water bridge (Figure 5B and Movie S6, Supporting Information). The regime maps (Figure 5C–E) plot success and failure in skimming an MP ball versus the ball diameter normalized by pitch distance, d/P, and the lifting speed normalized by the capillary velocity of σ/µ as Ca = V/(σ/µ), where µ is the water viscosity and Ca is usually referred to as the capillary number.^[^ ^40^, ^41^, ^42^ ^]^ Please note that the pitch and height were set to be the same (P = H) in this experiment; the results for P ≠ H will be discussed later (**Figure** **6**A). A total of fifteen types of MP balls were used in the experiment, which consisted of six different EPS foam balls and nine different balls made by 3D printing using polylactic acid (PLA) with varying infill densities (Table S1, Supporting Information). The density ratio refers to the ratio of water density to ball density. When the density ratio was very low (<0.1), skimming success was independent of the capillary number and determined only by the normalized ball size (Figure 5C). When the density ratio was in the intermediate range of 0.4–0.7, increasing Ca led to skimming failure, even when the normalized ball size was small (Figure 5D). When the density ratio was high in the range of 0.7–0.8, skimming failure was observed even with a smaller Ca (Figure 5E). This is because as the normalized lifting speed increased, the ratchet entrained more water as it exited the free surface of the water, thereby preventing the cutting of a lower water meniscus and consequent capture of an MP ball due to insufficient time for drainage.

### 2.5 Skimming MPs in Various Morphology

MPs come in a range of shapes and materials due to their origins from diverse sources, including fishing gear and single use plastics.^[^ ^43^, ^44^, ^45^, ^46^ ^]^ Based on our observations of marine debris during fieldwork near a port in Busan of South Korea (Figure S9A, B, Supporting Information), MPs were categorized into four groups: fragments, fibers, EPS foams, and pellets, as shown in Figure S9C (Supporting Information). To assess whether the hydrophilic ratchet can handle these different types of MPs, their skimming interactions were investigated as sequentially shown in Figure S10 (Supporting Information). Here, it is interesting to note that experiments conducted with other irregularly shaped plastic pellets, including a PE pellet (Figure S10B, Supporting Information) and a rod‐shaped PE pellet (Figure S10C, Supporting Information), showed similar skimming behaviors to that observed with the EPS ball (Figure S10A, Supporting Information), except for the case of a long Polyester fiber (Figure S10D, Supporting Information). This is mainly because the shape of meniscus around these MPs and their behavior did not vary much regardless of the specific morphology of the MPs. Furthermore, it was observed that the meniscus cutting also worked in the same manner as it did with the EPS ball, assisting in a more stable skimming of MPs. For the case of long fibers, it was found that the fiber was positioned vertically on the meniscus rather than horizontally, creating a water bridge that generates the Laplace pressure, thereby promoting adhesion (Figure S10D, Supporting Information). Based on these skimming interactions between irregular MPs and hydrophilic ratchet tooth, skimming was consistently observed successful for these MPs.

### 2.6 Extension of the Ratchet Structure

An analysis was conducted on ratchets with equal values for pitch and height (P = H) and was also extended to cover various forms of ratchets. The regime map (Figure S11A, Supporting Information) plots the skimming capability of ratchets when the height and pitch of the ratchet teeth are no longer the same (P ≠ H) but are still symmetric. d/H and d/P denote the ball diameter (d) normalized by ratchet tooth height (H) and by pitch (P), respectively. The capillary number (Ca = 1.4 × 10^−4^) and the density ratio (ρ_s_/ρ_l_ = 0.76) were set to be constant as control variables. MP balls could be easily skimmed off as long as their size was sufficiently small (d/H < 1 & d/P < 1) (Figure S11A, iii, Supporting Information). However, when the ball size increased to be comparable to H or P, the pitch distance could affect the skimming capability more easily at a smaller critical value (d/P = 1) than the ratchet tooth height (d/H = 1.5). Skimming failed in cases where the pitch was sufficiently large, but the height was too small (Figure S11A, i, Supporting Information), both the height and pitch were small (Figure S11A, ii, Supporting Information), or the height was sufficiently large but the pitch was small (Figure S11A, iv, Supporting Information). A skimming capability regime map was plotted using *asymmetric* ratchets with equal pitch and height (P = H) (Figure S11B, Supporting Information). The types of asymmetric ratchets included an asymmetric ratchet with the tip at the top (AR_t_, tip position = 1) (Figure S11B, i), Supporting Information), a symmetric ratchet with the tip at the middle (SR, tip position = 0.5) (Figure S11B, ii), Supporting Information), and an asymmetric ratchet with the tip at the bottom (AR_b_, tip position = 0) (Figure S11B, iii), Supporting Information). The geometries of AR_t_ (Figure S11B, i), Supporting Information) and SR (Figure S11B, ii), Supporting Information) maintained skimming capability, while AR_b_ (Figure S11B, iii), Supporting Information) showed a significant decrease in skimming capability. The lower surface of the ratchet also served as a physical support against gravity, and the magnitude of the force supporting it physically varied depending on the angle of the lower surface. As the angle of the lower surface increased from the free surface of water, the MPs received less physical support and were more easily swept away. We confirmed that physical support prevented the capillary water bridge from rupturing during skimming.

### 2.7 Tilt for Skimming High‐Density MPs

The skimming of MPs was affected not only by their size and mass, but also by their density. The low‐density MPs (0.02–0.80 g/cm^3^) formed concave menisci in their surroundings and were attracted toward the concave water meniscus formed on the hydrophilic plate. However, MPs such as PP and polyethylene (PE) have higher density (0.88–0.97 g cm^−3^ in pellets) than low‐density MPs (Table S2, Supporting Information).^[^ ^21^, ^47^, ^48^, ^49^ ^]^ As the MP pellets became heavier, a convex water meniscus formed around them, causing the pellets to no longer move toward the flat plate surface with a concave water meniscus due to the repulsive Cheerios effect (Figure S12A; see Supporting Information for details). On the other hand, when the plate was tilted at a positive angle, the repulsive force caused by the concave water meniscus curvature was reduced, releasing the repulsive Cheerios effect. When the plate was tilted at a positive angle, the 4‐mm PE pellet was able to freely approach the plate (Figure S12B, Supporting Information). However, for the ratchet plate (P = H = 6 mm) with a positive tilting angle, it was still impossible to skim the PE pellet vertically due to the repulsive Cheerios effect (Figure S12C and Movie S7, Supporting Information). This is because as the ratchet plate was lifted, it periodically formed a concave meniscus. In the end, the pellet could be skimmed when the ratchet plate was tilted at + 30° (Figure S12D and Movie S8, Supporting Information). Figure S11E (Supporting Information) shows the skimming capability based on the pitch (P = H) of the ratchet compared to the tilting angle. It was confirmed that a ratchet plate with a pitch and height greater than 6 mm could skim when tilted at + 30° or more.

### 2.8 Release of Capillary Adhesion

Once MP balls were lifted on the plate with a ratchet (P = H = 3 mm), they were released from the plate, and the capillary adhesion of a 2‐mm MP was reduced (Figure 6A). Immediately after release, the MP was observed to undergo horizontal drift in the opposite direction of the ratchet (36 ms). The PIV data (Figure 6B) show that water flow was induced in a direction to push the MP away from the ratchet during submersion into the water. The temporal evolution of water flow velocity, which was spatially averaged for the lower (Figure 6C) and upper (Figure 6D) sections of the ratchet tooth, reveals that water was introduced at a speed of ≈60 mm s^−1^ into the lower section of the ratchet (30–60 ms). Upon touching the free surface of water, the lower water meniscus became thicker due to the intake of water into the ratchet (Figure S13, Supporting Information). Subsequently, the water flow pushed the MP away from the ratchet, with positive u and v components of the flow at ≈53 ms (Figure 6D).

### 2.9 Theory for MP Adhesion on Flat Plates

**Figure 7:**
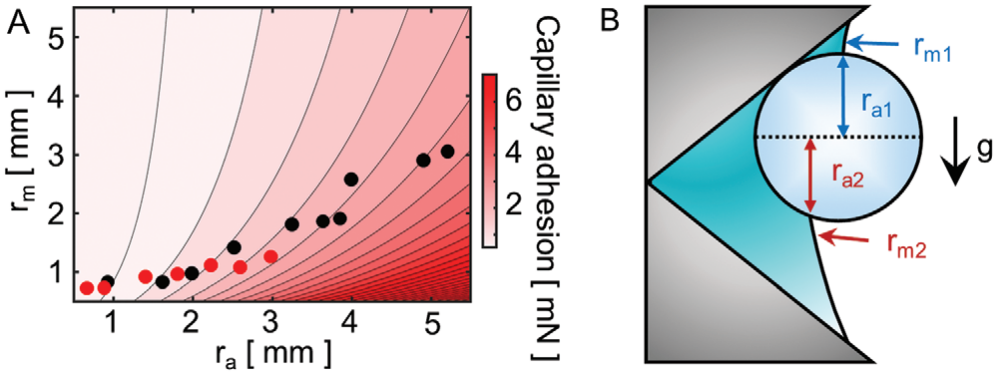
Estimation of capillary adhesion on an MP ball. (A) Contour plot of capillary adhesion with respect to r_a_ and r_m_. The black dots denote capillary adhesion on a flat plate, calculated based on measurements of r_a_ and r_m_ (Figure 3B, i). The red dots represent capillary adhesion of an MP ball in a ratchet with (B) an asymmetric configuration.

During the skimming process, an MP ball can be lifted and held due to capillary adhesion by the symmetric water meniscus between the ball and flat plate (Figure 3B, i); this process can be mathematically described by^[^ ^50^, ^51^, ^52^ ^]^;

(1) $$F_{\mathrm{ca},\mathrm{flat}}=\pi r_{a}\sigma \left(1+\frac{r_{a}}{r_{m}}\right)$$

where σ is the surface tension coefficient and *r*_a_ and *r*_m_ are the azimuthal and meridional radii of the curvature of the water bridge, respectively (Figure 3B, i). In Equation (1), the first term is the capillary pressure dictated by the curved water bridge: more capillary adhesion occurs when *r*_m_ is curved more concavely for the water phase (yielding a positive value of −π*r*_a_^2^∆*p* with *r*_a_ > *r*_m_, where *p* is the Laplace pressure). The second term quantifies the contact line resistance acting at the three‐phase contact line on the ball surface. The contour plot of capillary adhesion was drawn with respect to *r*_a_ and *r*_m_ (**Figure** **7**A); the black dots refer to the capillary adhesion data on a flat plate, with values calculated to be in the range of 0.5–3.5 mN by Equation (1). The estimated value of capillary adhesion was also similar to the normal‐holding data (1.0 ± 0.1 mN) (see Figure 3A). Besides capillary adhesion, other interactions such as electrostatic forces, π–π stacking, and pore‐filling mechanisms between the MPs and the ratchet structure might also be considered influential.^[^ ^53^ ^]^ For a discussion of their contributions, please see Section S4 (Supporting Information).

When gravity acted parallel to the plate surface (Figure 3B, ii), the maximum weight significantly dropped to 0.4 ± 0.1 mN (parallel‐holding data in Figure 3A). This observation serves as clear evidence that normal adhesion and shear adhesion resulting from the capillary water bridge exhibit distinct behavior. This is because sliding can occur without breaking the water bridge. If the shear adhesion on a flat surface is solely given by this contact line resistance of contact angle hysteresis, it can be described as^[^ ^54^, ^55^ ^]^:

(2) $$F_{\mathrm{hys},\mathrm{flat}}=2r_{c}\sigma \left(\cos\theta_{r}-\cos\theta_{a}\right)$$

where r_c_ is the contact radius of the water bridge on the flat plate and θ_r_ is the receding contact angle of water on the flat plate. Equation (2) then predicts that ≈0.001 mN will be the critical weight with *θ*_a_ in the range of 1–5° and θ_r_ = 0° when the shear adhesion is only determined by the contact angle hysteresis. However, this estimate was still lower than the values obtained from both the holding and skimming experiments, implying that there were other factors enhancing shear adhesion beyond contact angle hysteresis.

### 2.10 Theory for MP Adhesion to a Ratchet

*F*_ca,flat_ (Figure 3B, i) and *F*_hys,flat_ (Figure 3B, ii) were calculated based on the symmetric water bridge formed around an MP ball on a flat plate, which allowed the use of a single value for *r*_a_ and *r*_m_ in the equations. However, the capillary adhesion acting at a ratchet requires the consideration of asymmetric upper and lower water bridges (Figures 1 and 7B) with two sets of *r*_a_ and *r*_m_ values for each. Then, the capillary adhesion at the ratchet can be described as

(3) $$F_{\mathrm{ca},\mathrm{ratchet}}=\frac{1}{2}\;\pi r_{a1}\sigma \left(1+\frac{r_{a1}}{r_{m1}}\right)+\frac{1}{2}\pi r_{a2}\sigma \left(1+\frac{r_{a2}}{r_{m2}}\right)$$

which estimates intermediate values of the capillary adhesion acting at each upper and lower part of the water bridge. Equation (3) yields an estimate comparable to that for *F*_ca,flat_ from Equation (1) (Figure 7A), which is consistent with the measured data (Figure 3A). This allows the ratchet to maintain robust capillary adhesion even during skimming, as it did in the normal‐holding experiment.

### 2.11 Ratchet Drum for Continuous Recovery of Floating MPs

**Figure 8:**
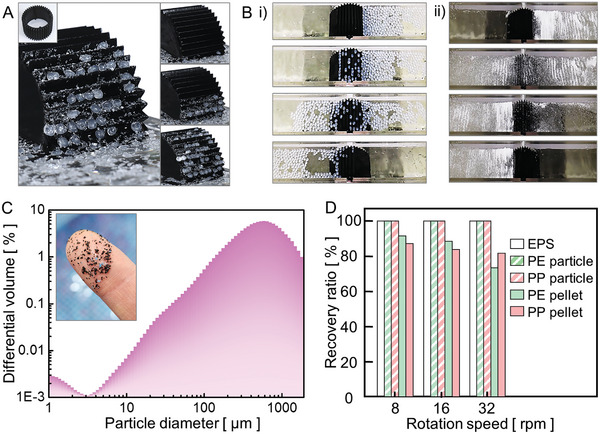
A prototype of a water‐bridged ratchet drum for different types of MPs. (A) The experimental images show MP skimming using a ratchet‐structured drum with P = H = 6 mm and a drum diameter of 60 mm. The drum skims a mixture of millimeter‐scale PE pellets and micrometer‐scale PE particles. (B) The ratchet‐structured drum is shown to remove all the MPs originally located on the right side of the container, and subsequently release them to the left side, irrespective of whether the MPs are i) PE pellets with a size of 2–4 mm, or ii) PE particles (C) ranging from 1 to 2000 µm. (D) Recovery ratios of the ratchet‐structured drum for five different MPs at three different drum rotation speeds.

Continuous recovery of floating MPs from the water surface was achieved by rotating a drum with a ratchet, collecting a floating mixture of millimeter‐scale PE pellets and micrometer‐scale PE particles (**Figure** **8**A). The drum had a diameter of 60 mm and a width of 50 mm, and the pitch and height of the ratchet were both 6 mm. The recovery of various MPs was tested by counterclockwise rotation of the ratchet drum in a rectangular tank with a width of 50 mm (Figure S14C, Supporting Information). The sequential images (Figure 8B, i) show the recovery of PE pellets with sizes of 2–4 mm as the floating MPs (right) were skimmed and transferred to the collecting container by releasing the skimmed MPs (left). Under the same conditions, sequential images are also shown for the recovery process of PE particles with sizes of 1–2000 µm (Figure 8B, ii and 8C and Movie S1, Supporting Information). It can be considered that most of the MPs were collected and transferred by the ratchet drum to the container regardless of shape or size. To calculate the recovery ratio, the MPs weight recovered on the left side of the drum was divided by the MPs weight initially introduced on the right side. The recovery ratio was measured by varying the rotational speed of the ratchet drum for EPS foam balls, PE pellets, PE particles, PP pellets, and PP particles (Figure 8D, Movies S1 and S9, Supporting Information). The EPS foam balls, and micrometer‐scale particles were recovered well even with an increase in the rotation speed of the ratchet drum. As the rotational speed increased, the amount of skimming for millimeter‐scale pellets decreased. This is because the shape of the water bridge was shifted from concave to convex and the slope of the water meniscus became steeper when more water was entrained at higher rotation speeds (Figure S15, Supporting Information). However, up to a rotational speed of 16 rpm, a recovery ratio of 80% or more was observed for all five types of MPs.

Additionally, tests were conducted to evaluate the performance of the hydrophilic ratchet drum under varying environmental conditions, such as waves, temperature, and wind. For more details, see Section S5 (Supporting Information).

### 2.12 Skimming of Weathered MPs

Previous studies have reported that the primary change noted as MPs endure prolonged exposure to a marine environment is hydrophilization with the development of biofilms.^[^ ^56^, ^57^, ^58^, ^59^ ^]^ Experiments were conducted to investigate how the interaction between the hydrophilic ratchet and MPs changes as the surface properties of the MPs become more hydrophilic, first by hydrophilizing PP ball and then with non‐circular PE pellets that were weathered in seawater with phytoplankton and viscous oil of low sulfur fuel oil (LSFO). Here, LSFO is the mandated fuel oil for use on aboard ships, in compliance with current IMO regulations regarding low sulfur content.^[^ ^60^ ^]^ Figure S18A (Supporting Information) shows the hydrophilization of originally hydrophobic PP ball from the water CA of 100 ± 5° to 20 ± 5° after the oxygen plasma treatment. Following this hydrophilization, the floating state of the PP ball changed as shown in Figure S18B (Supporting Information). Initially, it partially sank with a convex meniscus, but after being hydrophilized, it submerged further, exhibiting a concave meniscus. This increase in the concavity of the meniscus helped the hydrophilized PP ball move closer to the ratchet through the Cheerios effect. Figure S18C (Supporting Information) shows the sequential images presenting similar capillary skimming behaviors between untreated (Figure S18C, i, Supporting Information) and hydrophilized (Figure S18C, ii, Supporting Information) PP balls when they were skimmed by a ratchet‐structured hydrophilic plate (P = H = 6 mm). In both instances, skimming was observed to be successful, as the hydrophilicity of MPs would promote the skimming process.

**Figure 9:**
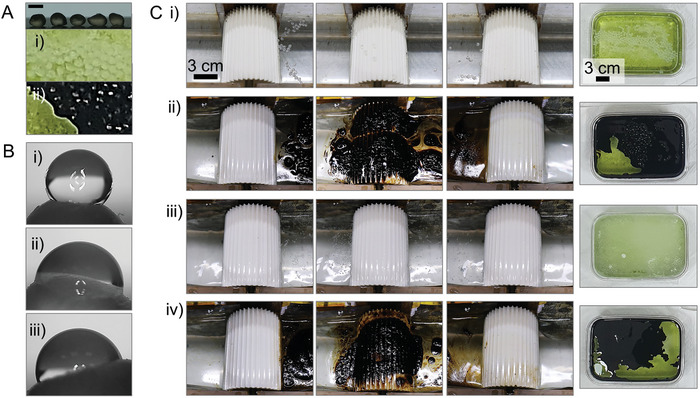
Skimming MPs weathered in seawater with phytoplankton and oil. (A) Non‐spherical PE pellets subjected to weathering as immersed in seawater for a week with i) phytoplankton alone and ii) both of phytoplankton and low sulfur fuel oil (LSFO). The scale bar is 3 mm. (B) The changes in water contact angle after one week of weathering, indicating that i) a pristine PE pellet was hydrophilized in these weathering conditions: ii) phytoplankton alone and iii) both of them. (C) The sequential images of the drum skimming of PE pellets weathered in seawater with i) phytoplankton alone and ii) both (the far‐right images). The skimming experiments were also performed with smaller PE particles under these two weathering conditions of iii) phytoplankton alone and iv) both. The far‐right images show the weathering of MPs in each condition.

**Figure** **9**A–C present other experiments that were conducted with weathered PE pellets. Please note that PE pellets were used here, instead of PP balls, to perform the experiment with non‐spherical MPs rather than spherical PP balls. After subjecting PE pellets to weathering in an environment with seawater (salinity ≈ 34‰) and phytoplankton (chlorella) for one week (Figure 9A, i), the water CA of PE pellet decreased from 95 ± 5° to 70 ± 10° (Figure 9B), which indicates hydrophilization.^[^ ^52^, ^54^ ^]^ Figure 9C, i) shows that the skimming experiment for the weathered PE pellets using a hydrophilic ratchet drum was conducted successfully. Once again, this is because an increase in the hydrophilicity of MPs would promote skimming performance. Additionally, another weathering experiment was conducted to simulate an oil spill environment by subjecting PE pellets to weathering in seawater for one week including an organic contaminant, phytoplankton and a fuel oil of LSFO (Figure 9A, ii). It was shown that the hydrophilic ratchet drum successfully skimmed the PE pellets fouled by these two contaminants (Figure 9C, ii). In skimming such contaminated MPs, it was again observed that the meniscus cutting played a role in preventing the upper meniscus from being overloaded with too many MPs, as shown by the side view images in Figure S19 (Supporting Information). It is also worth noting that the hydrophilic ratchet remained relatively clean even after the experiment with LSFO that is highly adhesive with a viscosity of 2360 cSt. This indicates that the releasing mechanism of the hydrophilic ratchet discussed earlier was quite effective, even capable of detaching such highly viscous oil. Next, identical weathering experiments were performed for smaller MPS ranged from 1 µm to 2 mm in size, successfully skimming the contaminated MPs of various sizes (Figure 9C, iii, iv).

In addition to the weathering of MPs, the long‐term performance of the hydrophilic ratchet was also assessed through biofouling experiments. The hydrophilic ratchet was submerged in seawater for seven days, and the results showed that the superhydrophilic surface effectively prevented biofilm attachment, with no observed performance degradation. SEM images confirmed that no significant biofilm formation occurred on the surface after the 7‐day experiment (Figure S20, Supporting Information).

### 2.13 Scale‐Up Devices for Skimming Floating MPs in Open Marine Environments

**Figure 10:**
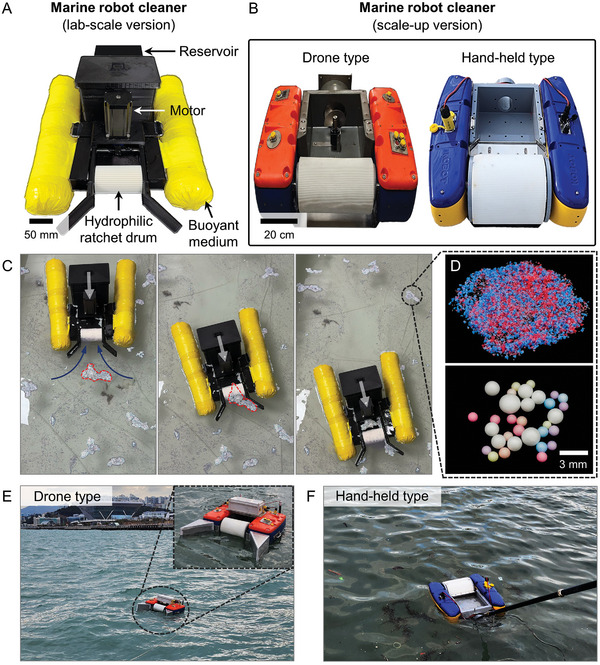
Prototypes of the marine robot cleaner equipped with a water‐bridged ratchet. Photo images of (A) lab‐scale and (B) scale‐up versions of the marine robot cleaner. (C) The sequential images showing that the lab‐scale prototype equipped with the front ratchet drum is skimming MPs of various sizes while moving forward (see Movie S10). (D) The MPs used in the skimming experiment in (C). (E) A drone‐type of marine robot cleaner for navigating the open sea, and (F) a hand‐held type for manually cleaning areas close to docks or seashore.

A marine robot cleaner equipped with the hydrophilic ratchet drum was developed as a proof‐of‐concept device for practical applications in marine environment (**Figure** **10**). The lab‐scale prototype of the marine robot cleaner is shown in Figure 10A, and its scale‐up versions (Figure 10B) are displayed in two distinct types: a drone (Figure 10E) and a handheld device (Figure 10F). The marine robot cleaner features a front‐mounted hydrophilic ratchet‐structured drum to collect floating MPs. It was equipped with buoyant components on both sides and propellers for navigating in open marine environments. The lab‐scale prototype had dimensions of 85 cm (length) × 42 cm (width) × 35 cm (height), with a total weight of 8 kg (Figure 10C). The remote control enabled the adjustment of the rotation speed of the ratchet drum, with a diameter of 6 cm and ratchet teeth (P = H = 4.5 mm), from 1 to 100 rpm, and the direction and speed of the propellers within a remote range of 10 m. Figure 10C and Movie S10 (Supporting Information) show that the front ratchet drum of the lab‐scale prototype was effectively skimming MPs (PE and PP particles, and EPS foam balls) with various sizes from 50 µm to 5 mm (Figure 10D) while moving forward. The forward propulsion from the propeller was also noted to be quite beneficial in collecting MPs dispersed across various locations in the open environment. Beyond the lab‐scale device, the scale‐up version of the marine robot cleaner was developed to regularly navigate the water surface for cleaning the MPs for a long‐term use in marine environments (Figure 10E), by enhancing the mechanical durability, waterproofing, as well as wireless controllability, battery life, and charging convenience. Additionally, the current technology for removing MPs floating on the water surface has a limitation in addressing MPs dispersed at various depths below the surface. To overcome this, we are exploring the integration of bubble flotation into the hydrophilic ratchet system, whereby bubbles are used to lift submerged MPs to the surface for removal. Future research will focus on optimizing bubble size and utilizing eco‐friendly surfactants to enhance the attachment of bubbles to MPs dispersed below the water surface in real marine environments.^[^ ^61^, ^62^ ^]^ We are also looking into mounting a device that generates water surface flow toward the drum like previous examples.^[^ ^63^, ^64^ ^]^ Besides the drone type, a hand‐held device akin to a household vacuum cleaner was designed to enables users to grip the handle and manually clean the MPs floating on water close to the docks or seashore (Figure 10F). This hand‐held device, being designed to be lighter than the drone type without propellers, heavy battery, and remote controller, is expected to offer a convenient manual solution for cleaning areas near docks. While the present work focused on removing floating MPs in open marine systems, the hydrophilic ratchet drum can also be extended to other challenging environments, such as surface runoff in water channels. Surface runoff often carries MPs into larger bodies of water, and addressing this issue at its source can significantly reduce overall marine pollution.^[^ ^65^ ^]^

The marine robot cleaner has several advantages in comparison to the conventional size‐based filtration technology like the manta net^[^ ^26^, ^27^, ^28^, ^66^, ^67^, ^68^ ^]^ (Table S3, Supporting Information). The size‐based filtration method is known to suffer from a significant drawback of clogging.^[^ ^26^, ^28^ ^]^ The clogging problem arises much more easily in marine environments where the diverse range of sizes of contaminants and sticky pollutants are present. Accordingly, the utilization of nets poses not only efficacy issues by clogging but also high maintenance demands associated with frequent net replacements. Compared to this, the hydrophilic ratchet presents distinct maintenance benefits as it is not susceptible to pore clogging and employs water bridges to reduce contact with contaminant. Furthermore, the hydrophilic ratchet can skim most of floating particles, such as MPs or NPs in various shapes and sizes, including the fibrous shape, which may escape through the net or mesh pores depending on their orientation. Overall, the hydrophilic ratchet device is believed to offer a new option for removing MPs with not only enhanced effectiveness but also operational benefits in open marine environments.

## 3 Conclusion

In this study, we introduced a novel capillary skimming mechanism utilizing a hydrophilic ratchet to remove floating MPs from the water surface. The proposed method exploits the Cheerios effect and the formation of a capillary water bridge to skim and securely hold MPs of varying sizes and densities. Our findings demonstrated that the hydrophilic ratchet outperforms flat in skimming performance, recovering MPs with sizes ranging from 1 µm to 4 mm and densities from 0.02 to 0.97 g cm^−^^3^. The implementation of the ratchet structure in a marine robot cleaner prototype further validated the practicality of our approach for large‐scale MP recovery. However, to operate in real‐world conditions, battery issues must be addressed, and we are exploring supplementary means such as solar panels. This capillary skimming method provides a significant advancement in addressing marine plastic pollution, offering a versatile and efficient solution for the removal of MPs from aquatic surfaces.

## 4 Experimental Section

### Sample Preparation and Characterization

The material used was polylactic acid (PLA), and the flat and ratchet‐shaped samples were fabricated using an FDM 3D printer (Single Plus, Cubicon, Republic of Korea). The 3D printer had a nozzle diameter of 0.4 mm, with a nozzle temperature of 215 °C and a bed temperature maintained at 60 °C during the printing process. To make the surface of the samples (plate‐type or drum‐type) hydrophilic, they underwent O_2_ ion beam treatment for 30 min to 1 hr in a vacuum chamber. Throughout the treatment, the chamber pressure was kept at 20 mTorr, the oxygen flow rate was set to 20 sccm, and the power was adjusted to 50 W. For uniform treatment of the cylindrical surface of the drum‐type samples, a rotating stage was employed during the O_2_ ion beam treatment.

### Holding and Skimming Experiments

The “holding” experiment was conducted by manually placing an MP ball on a plate, with a water meniscus between the two components, and observing whether the ball fell due to gravity. The “skimming” experiment, on the other hand, involved lifting a plate parallel to its surface to skim a ball from the free surface of water. The skimming experiment was controlled in a speed range of 1–50 mm s^−1^ using a linear stage (SL1‐1520‐4S, Science town, Republic of Korea).

### Particle Image Velocimetry

The flow near the surface during lifting or dipping of the plate was observed using the PIV method. Images were captured while lifting or dipping the plate, with a focus on the movement of TiO_2_ nanoparticles dispersed in water. A high‐speed camera was used to take images at a rate of 3000 frames per second (FASTCAM NOVA S9, Photron, Japan). The images were analyzed using PIVlab,^[^ ^69^ ^]^ and vectors were overlaid onto the original experimental images based on the analyzed results. A homemade source code is used for postprocessing. The TiO_2_ nanoparticles with a particle size of less than 50 nm were purchased from Sigma‐Aldrich.

### Microplastic Recovery Experiment by Ratchet Drum

A ratchet drum with a width of 50 mm and diameter of 60 mm (Figure 8A; Figure S13, Supporting Information) was employed to recover MPs. The ratchet drum recovered EPS foam balls, PE and PP pellets (2–4 mm), and PE and PP particles (1–2000 µm) (Table S2, Supporting Information). The recovery ratio was determined by dividing the weight of MPs collected on the left side of the drum by the weight of MPs initially introduced on the right side. The drum rotational speed was controlled at values ranging from 8 to 32 rpm. The total experiment duration was set to 5 min for all runs.

## Conflict of Interest

The authors declare no conflict of interest.

## Author Contributions

S.C., S.J.K., M.‐W.M.: conceptualization. S.C., S.J.P., S.J.K.: methodology. Y.A.L., H.‐Y.K., S.J.K.: investigation. S.C., Y.J.L., Y.J.L.: visualization. S.J.K., S.C., M.‐W.M.: supervision. S.C., S.J.K., M.‐W.M.: writing—original draft. S.C., H.‐Y.K., S.J.K., M.‐W.M.: writing—review & editing.

## Supporting information



Supporting Information

Supplemental Movie 1

Supplemental Movie 2

Supplemental Movie 3

Supplemental Movie 4

Supplemental Movie 5

Supplemental Movie 6

Supplemental Movie 7

Supplemental Movie 8

Supplemental Movie 9

Supplemental Movie 10

## Data Availability

The data that support the findings of this study are available in the supplementary material of this article.
