# Supplementary material for: Capillary Skimming of Floating Microplastics via a Water‐Bridged Ratchet
Source: Adv Sci (Weinh). 2024 Nov 5;12(1):2408623. doi: 10.1002/advs.202408623 (PMC11714184; doi:10.1002/advs.202408623)
Supplement: Supplementary file 1 — Supporting Information [file ADVS-12-2408623-s007.pdf]

ADVANCED SCIENCE
Open Access

Supporting Information

for *Adv. Sci.*, DOI 10.1002/advs.202408623

Capillary Skimming of Floating Microplastics via a Water-Bridged Ratchet

Seohyun Cho, Sang Jin Park, Young Jin Lee, You Jun Lee, Young A Lee, Ho-Young Kim, Seong Jin Kim, Seok Chung and Myoung-Woon Moon**

Supplementary Materials for

Capillary Skimming of Floating Microplastics via a Water-Bridged Ratchet

Seohyun Cho, Sang Jin Park, Young Jin Lee, You Jun Lee, Young A Lee, Ho-Young Kim, Seong Jin Kim*, Seok Chung and Myoung-Woon Moon*

*Corresponding author. Email: kyk756@kist.re.kr (S. J. K.); mwmoon@kist.re.kr (M.-W. M.)

This PDF file includes:

Supplementary Text
Figs. S1 to S22
Tables S1 to S3
Lists of supplementary movies from S1 to S10
References 1 to 89

Other Supplementary Materials for this manuscript include the following:

Movie S1 (.mp4 format). Collecting the various MPs using the hydrophilic ratchet drum.
Movie S2 (.mp4 format). EPS foam ball being skimmed by the hydrophilic ratchet plate.
Movie S3 (.mp4 format). Skimming small and large EPS foam balls using a hydrophilic flat plate.
Movie S4 (.mp4 format). Flow vector field generated while lifting the ratchet plate.
Movie S5 (.mp4 format). Flow vector field generated while lifting the flat plate.
Movie S6 (.mp4 format). Capillary water bridge ruptured and skimming failed.
Movie S7 (.mp4 format). The ratchet plate failed to skim a single PE pellet (P = H = 6 mm).
Movie S8 (.mp4 format). Tilting the ratchet plate at + 30° to skim a single PE pellet (P = H = 6 mm).
Movie S9 (.mp4 format). Skimming and release behavior of PP on the hydrophilic ratchet drum.
Movie S10 (.mp4 format). Marine robot cleaner equipped with a water-bridged ratchet for skimming microplastics.

Supplementary Text

S1. Capillary skimming with Cheerios effect

A solid object floating on water experiences the directional force from the surrounding curved capillary water meniscus (*35, 70−72*). This phenomenon has been studied in the context of water-walking insects, such as the larva of the waterlily leaf beetle Pyrrhalta, which climb on water meniscus by adjusting their body shape to take advantage of the directional force generated by the curved water meniscus (*73*). The phenomenon of directional force between two solid objects, which is generated by a curved liquid meniscus, is commonly referred to as the "Cheerios effect". This term originates from the behavior of Cheerios breakfast cereal floating in milk, which tend to clump together or adhere to the walls of the container. (*35*). The Cheerios effect has been used to explain a range of natural phenomena, such as the clustering of aquatic plants in certain areas due to the adherence of floating seeds to one another and to other plants or rocks (*74, 75*). It was observed that MPs floating on the water also exhibit interactions with one another and with surrounding object. The direction of the force is determined by the shape of the water meniscus formed between the two objects. Figure S21 shows the direction of the force depending on the shape of the water meniscus. When the menisci between the plate and the ball are formed by combining concave and concave shapes (magenta contour in fig. S21A), or convex and convex shapes (blue contour in fig. S21B), attractive force is generated. Conversely, when the menisci are formed by combining concave and convex shapes (fig. S21C), or convex and concave shapes (fig. S21D), the plate and the ball experience repulsion force. When the plate is tilted in the negative (−) direction, the height of the concave water meniscus increases, resulting in an increase in the magnitude of the repulsion force generated (fig. S21E). When the plate is tilted in the positive (+) direction, the height of the concave water meniscus decreases (red contour in fig. S21F), resulting in the disappearance of the repulsion force. In this way, the MP floating on water can receive directional force in various ways depending on the buoyancy of the MP and the angle of inclination of the plate (*70, 71, 76−78*).

The buoyancy of MPs could vary due to the wide range of shapes, densities, and sizes. The EPS foam balls and PE pellets were shown floating in water in a glass container (fig. S22A) and a PS container (fig. S22B), respectively. On the hydrophilic glass container wall, a concave water meniscus was formed, and the EPS foam ball was observed to form the concave water meniscus and contact the container due to the attraction force. Furthermore, EPS foam balls were observed to float at a distance from PE pellets, which formed convex water meniscus in their vicinity. As shown in Figure S22B, the formation of convex meniscus on the hydrophobic PS container wall indicated that the PE pellets were in contact with the container wall. We have confirmed that MPs, which form concave water meniscus in their vicinity, such as EPS foam balls, can assist in capillary skimming by providing attraction force when utilizing hydrophilic surface. MPs with density similar to that of water, such as PE and PP pellets, were found to experience a repulsive force near the concave water meniscus formed on the hydrophilic surface, resulting in no contact with the plate surface. Even on a hydrophilic surface, tilting in the + direction resulted in the formed concave water meniscus being almost identical to the free surface of water, thereby eliminating the repulsion force generated by the Cheerios effect (fig. S12B). Eventually, the PE pellets were capillary skimmed by tilting the ratchet plate (fig. S12D).

S2. Skimming of agglomerated MPs

An experiment to skim two different types of MPs (EPS foam balls and PE particles) using a hydrophilic ratchet plate as shown in fig. S7. The EPS balls were between 1.5 mm and 2.0 mm in

size, while the PE particles were smaller than the EPS balls as shown by the size distribution, whose peak was at 0.6 mm, of fig. S7C. Figure S7A shows that these MPs was successfully skimmed with the meniscus cutting by the ratchet tip which separated MPs each other. This meniscus cutting was also observed to help skim agglomerated MPs by preventing the upper meniscus from being overloaded with too many MPs. This characteristic can be more clearly presented when a small 1.5 mm EPS ball was skimmed alongside a larger 4 mm EPS ball, as sequentially shown in fig. S7B. It is shown that the ratchet valley (defined by the adjacent two tooth tips) initially took the smaller 1.5 mm ball, then this smaller ball was separated from the larger one. After that, the next ratchet valley skimmed the larger 4 mm ball. This separation behavior by the ratchet teeth prevented one single ratchet valley that was already holding a 1.5 mm ball from becoming overloaded with an additional 4 mm ball.

S3. Rotation speed of the ratchet drum and the water bridge

Figure S15 shows that the thickness of water bridge became thicker with an increased amount of entrained water while the rotation speed increased from 8 rpm to 128 rpm, corresponding to line speeds of 3 cm/s and 47 cm/s, respectively, given the drum diameter of 7 cm. This is presumably because the geometry of the ratchet structure contributed to scooping more water out of its surface, overcoming gravity as the lifting inertia increased with faster rotation. An interesting observation is that as the rotation speed increased from 8 rpm to 32 rpm, the shape of the water bridge shifted from concave to slightly convex. Furthermore, as the rotation speed continued to rise to 64, 96, and finally 128 rpm, the water bridge became thicker and thicker along with an increase in convexity. Besides the water bridge, the slope of the water meniscus became steeper as more water was entrained with faster rotation speed. Consequently, at higher rotation speeds, MPs flowed down along this meniscus as if sliding on a slope. Additionally, in fig. S15, the yellow arrows denote a shift to higher location of meniscus cutting as the rotation speed increased. Accordingly, MPs were subjected to the downward pulling effect from the water meniscus for extended locations. As a result of the effects outlined above, MPs were skimmed only at relatively low rotation speeds up to 32 rpm.

S4. Mechanisms of interaction between microplastics and the ratchet in capillary skimming

The interactions between MPs and the ratchet can be explained through electrostatic interactions, π–π stacking, pore-filling, and diffusion mechanisms, all of which are influenced by environmental conditions (53). Electrostatic forces, such as those from zeta potential, can affect the behavior of MPs in solution, but their impact is relatively minor compared to capillary forces. The Cheerios effect is governed by the deformation of the water meniscus and capillary action, which minimizes the influence of electrostatic repulsion or attraction between MPs, as the magnitude of electrostatic forces is significantly smaller than that of capillary forces. Additionally, π–π interactions and pore-filling mechanisms are important for chemical interactions between MPs and organic pollutants in environmental contexts. However, in capillary skimming, the movement of MPs is primarily driven by physical forces, such as surface tension, rather than molecular-level interactions like π–π stacking. Factors like pH, salinity, and dissolved organic matter can modify the surface characteristics of MPs, but their influence on capillary skimming is secondary to surface tension, which remains the dominant factor in this mechanism.

S5. Environmental impact on hydrophilic ratchet drum performance in skimming microplastics

Tests were conducted to evaluate the performance of the hydrophilic ratchet drum under varying environmental conditions, such as waves, temperature, and wind. The results showed that while the skimming performance was not significantly affected by moderate wave heights (fig. S16A), larger waves could submerge the water bridge, reducing the recovery efficiency of MPs (fig. S16B). When the water temperature was controlled at 6°C, it was confirmed that the skimming performance was not affected, as the change in surface tension due to temperature was minimal (fig. S17A). Wind blowing in the skimming direction enhanced performance (fig. S17B), but when blowing in the opposite direction, MPs located farther away could not be captured (fig. S17C). These findings suggest that the ratchet system performs robustly under moderate conditions, but further research is needed for real-world applications.

S6. Challenges and cost benefits of scaling hydrophilic ratchet technology for ocean cleanup

While the hydrophilic ratchet-based system shows promising results, scaling up this technology for widespread implementation across large oceanic regions may pose economic challenges. Initial investments for robotic cleaning systems equipped with this technology could be high, particularly in mobile applications (Table S3). However, the costs associated with conventional net-based methods, which require frequent vessel operations and maintenance, suggest that our system may offer cost advantages in the long run by reducing operational expenses. Furthermore, as autonomous surface robots are becoming more common in marine management, the integration of the hydrophilic ratchet system may complement future advancements in smart ocean-cleaning technologies.

S7. Mitigating environmental impacts in field applications of hydrophilic ratchet technology

Field studies are necessary to evaluate the unintended recovery of other marine particles, suspended solids, and organisms that may affect the marine ecosystem. Strategies such as avoiding sensitive areas or incorporating separation technologies will be crucial to minimizing potential environmental impacts.

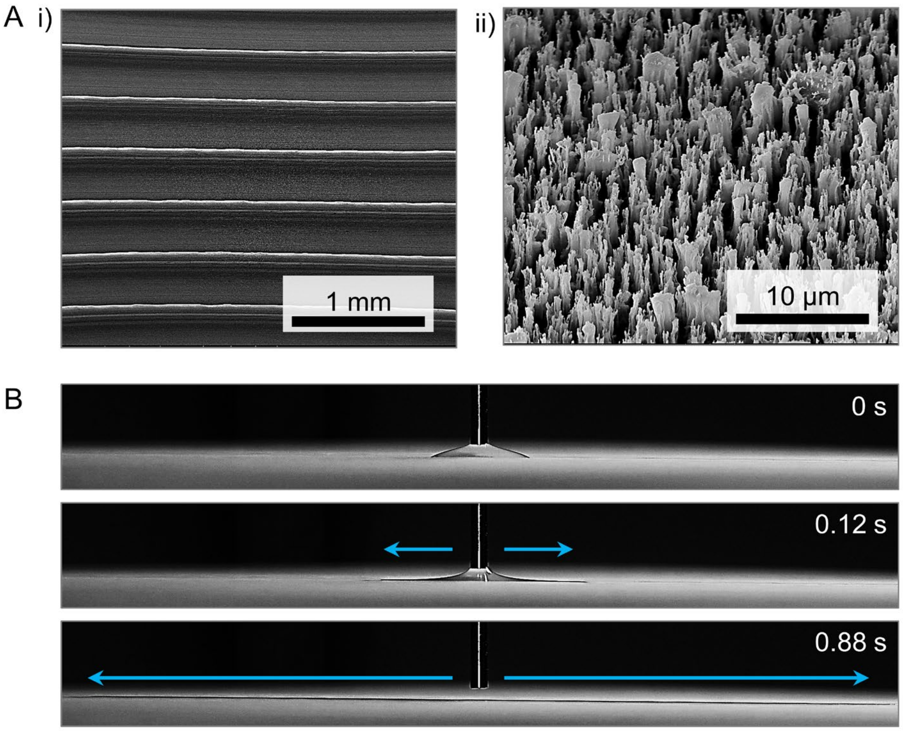

Fig. S1. Surface characteristics of oxygen plasma treated Polylactic Acid (PLA). (**A**) SEM images of the oxygen plasma treated surface of PLA. (i) 100X, showing the rastered line pattern by a 3D printer and (ii) 10,000X, showing a high-aspect-ration nanostructures formed by oxygen plasma etching. (**B**) Optical images showing the sequential behavior of the water spreading behavior on the hydrophilic PLA surface.

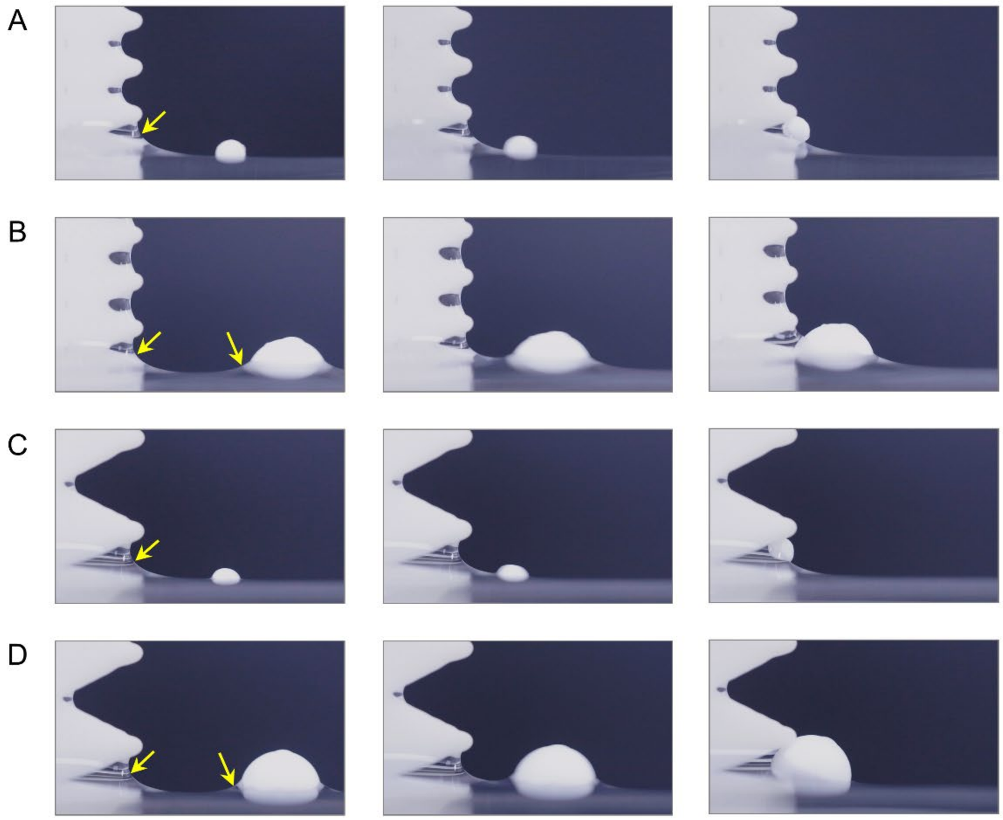

Fig. S2. Experimental images showing the critical distance at which the Cheerios effect is activated under various conditions. (A) A 2 mm EPS foam ball and ratchet teeth with P = H = 3 mm. (B) A 5 mm EPS foam ball with ratchet teeth of the same size. (C) A 2 mm EPS foam ball with larger ratchet teeth, P = H = 6 mm. (D) A 5 mm EPS foam ball with ratchet teeth of the same size.

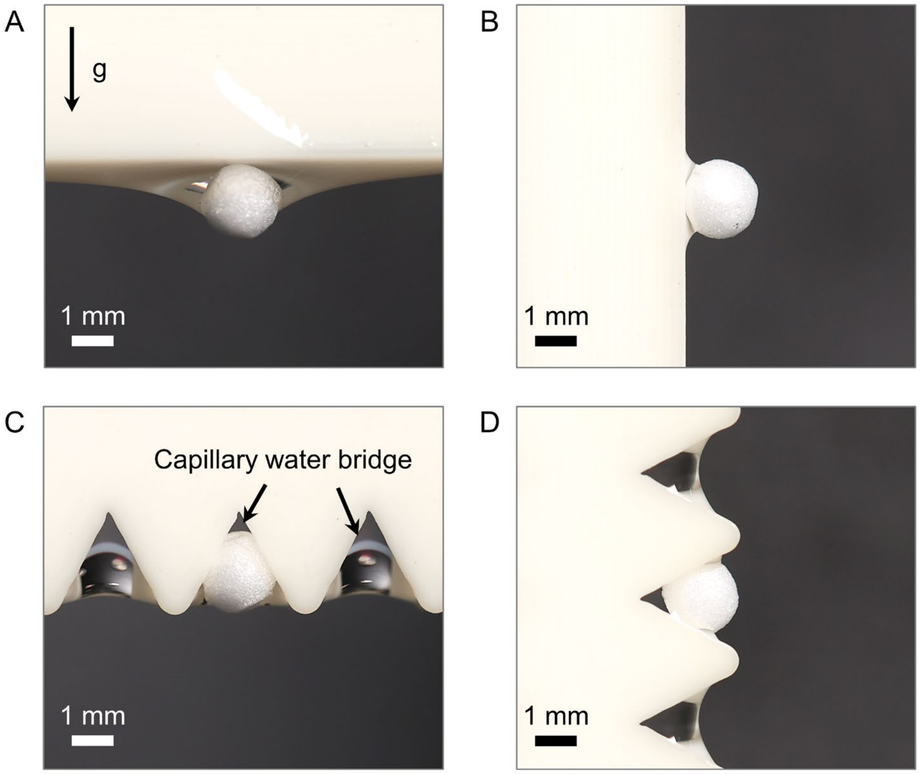

Fig. S3. Formation of a stable capillary adhesion with a water bridge. Optical images showing EPS foam balls held on (**A**) the flat plate normal to gravity and (**B**) the flat plate parallel to gravity. Optical images showing the EPS foam ball held on (**C**) the ratchet plate normal to gravity and (**D**) the ratchet plate parallel to gravity. Arrows indicate the capillary water bridge formed between the ratchet tooth.

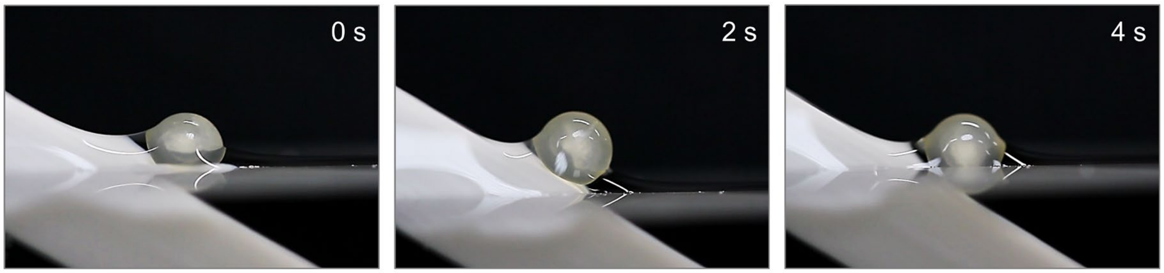

Fig. S4. Sequential images of failed attempts to skim a 3-mm polypropylene (PP) ball using a flat plate tilted at + 60°. For the initial 2 s, the PP ball adhered to the plate and lifted before sliding down as the upper water bridge receded (4 s).

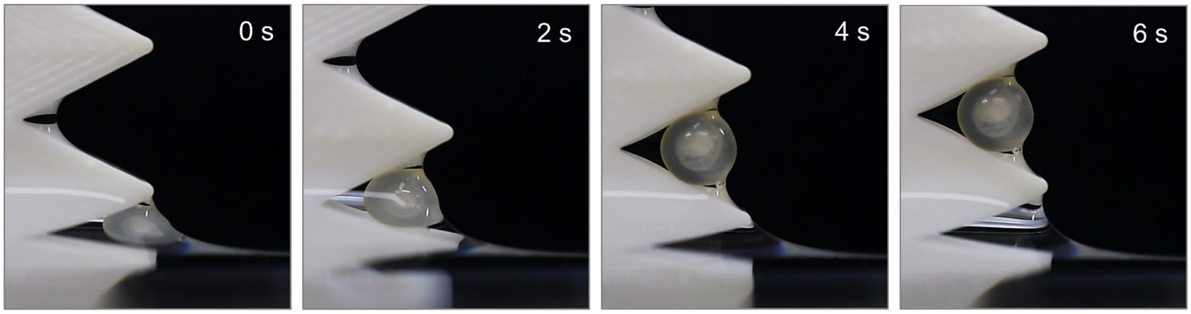

Fig. S5. Sequential images of capillary skimming a 3-mm polypropylene (PP) ball using a ratchet structure. The water bridge formed around the PP ball, maintaining stable capillary adhesion.

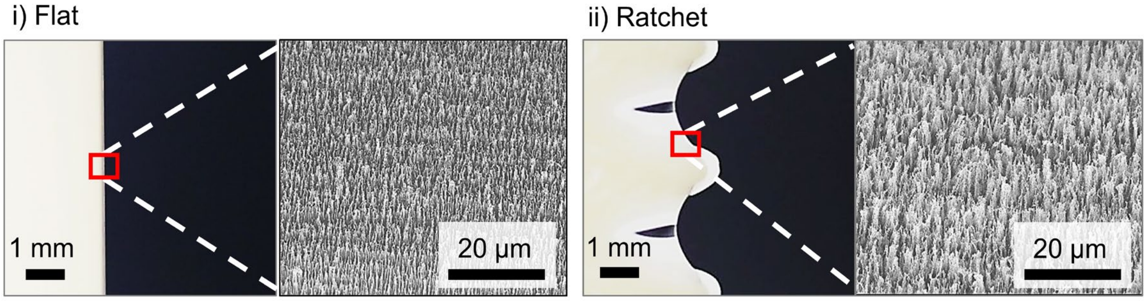

Fig. S6. Surface characteristics of Polylactic acid (PLA) with ratchet structure. An optical image of the plate and a SEM image of the indicated area captured at a higher magnification (5,000X), i) the flat plate, ii) the ratchet plate in the side view. All surfaces are hydrophilic, with the ratchet in particular being capable of containing a significant amount of water internally.

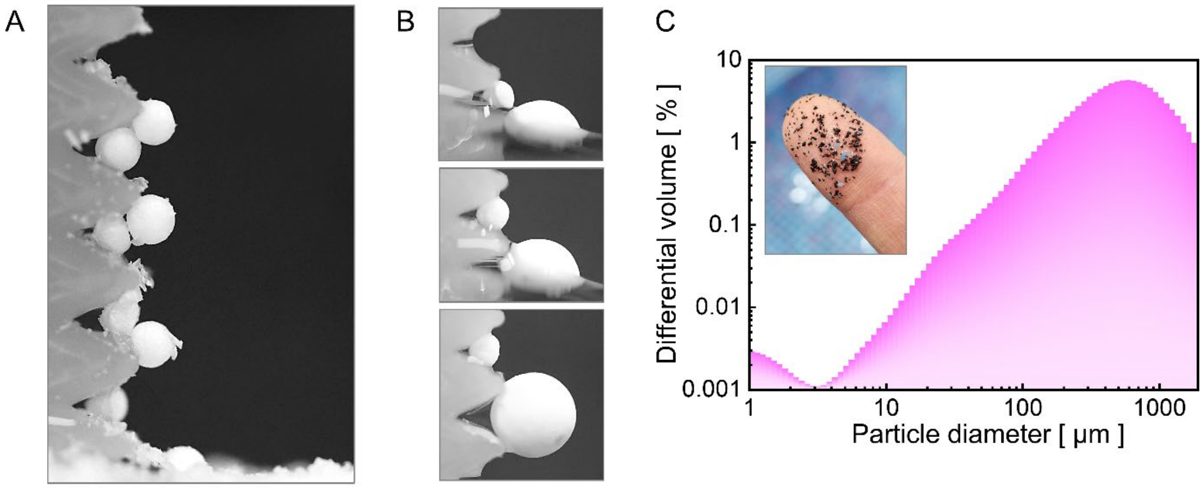

Fig. S7. Capillary skimming with a mixture of particles of various sizes. (**A**) The side-view of skimming two different types of MPs (EPS foam balls and PE particles) using a hydrophilic ratchet plate (P = H = 3 mm). The EPS balls (1.5 – 2.0 mm in size) were larger than the PE particles whose size distribution with a peak at 0.6 mm is plotted in C. (**B**) The sequential images of skimming two neighboring MPs with different sizes of 1.5 mm and 4.0 mm using the hydrophilic ratchet plate. (**C**) The size distribution of the MPs used in (**A**).

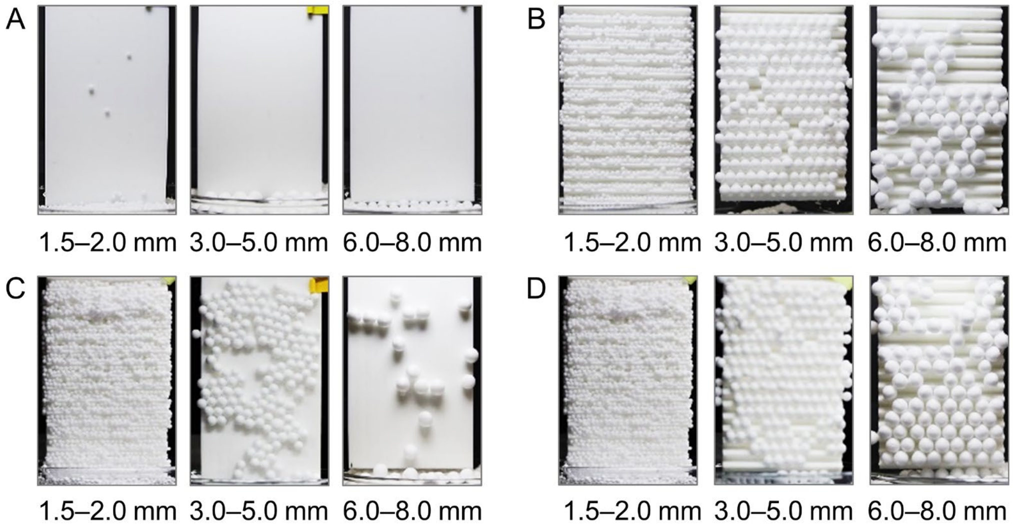

Fig. S8. Optical images of EPS foam balls skimmed on the four different plates with three different sets of the ball diameters (1.5−2.0, 3.0−5.0 and 6.0−8.0 mm in diameter). Optical mages in the front view showing the 4 different types of the skimming surfaces; (**A**) the hydrophobic flat, (**B**) the hydrophobic ratchet, (**C**) the hydrophilic flat, (**D**) the hydrophilic ratchet.

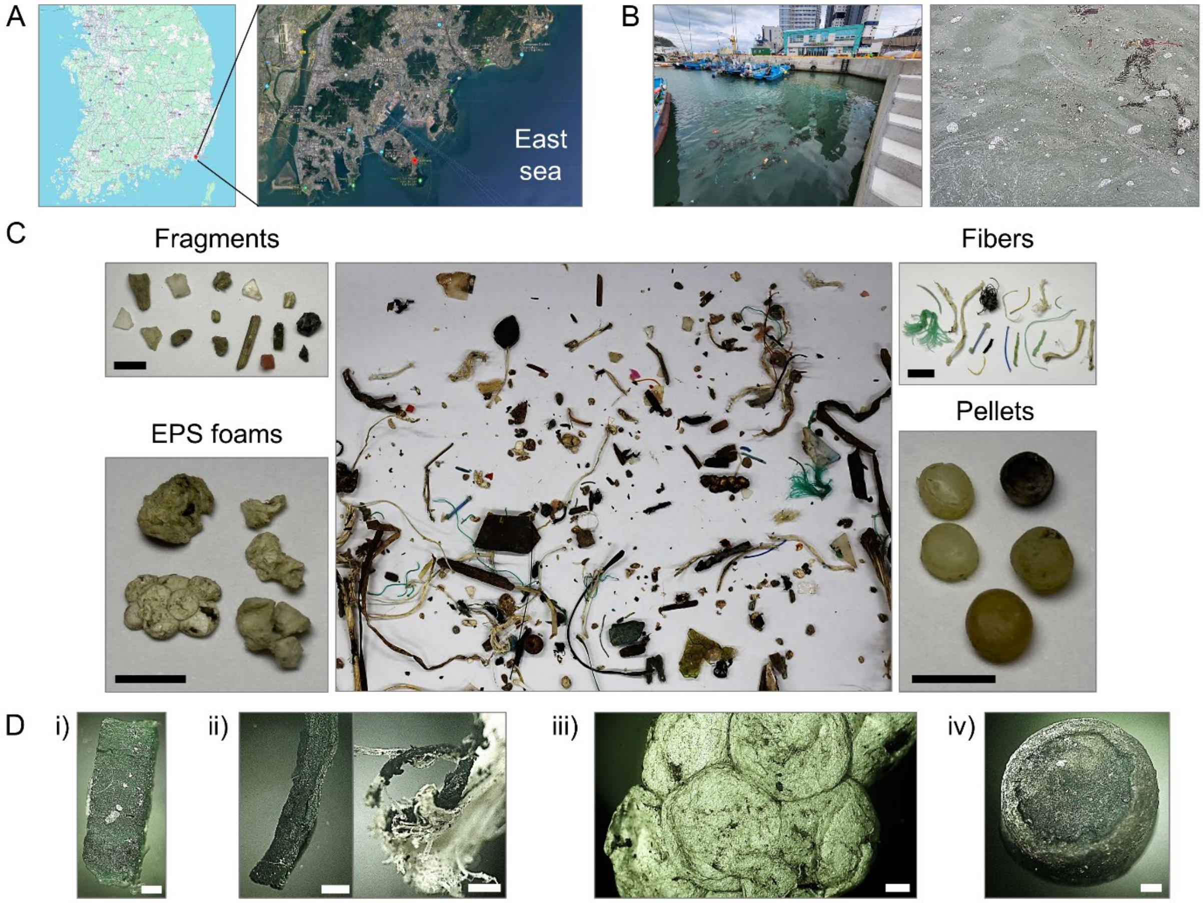

Fig. S9. MPs collected from a real marine environment. (**A**) MPs were collected from the bay area in Busan, a coastal city in the southeast region of the Korean peninsula. (**B**) Photo images of MPs and other marine debris floating near ports and coastal area. (**C**) The collected MPs were categorized into fragments, fibers, EPS foams, and pellets. The scale bars are 5 mm. (**D**) Optical images of MPs underwent bio-fouling in various types: i) fragment, ii) fiber, iii) EPS foam, and iv) pellet. The scale bars are 500 μm.

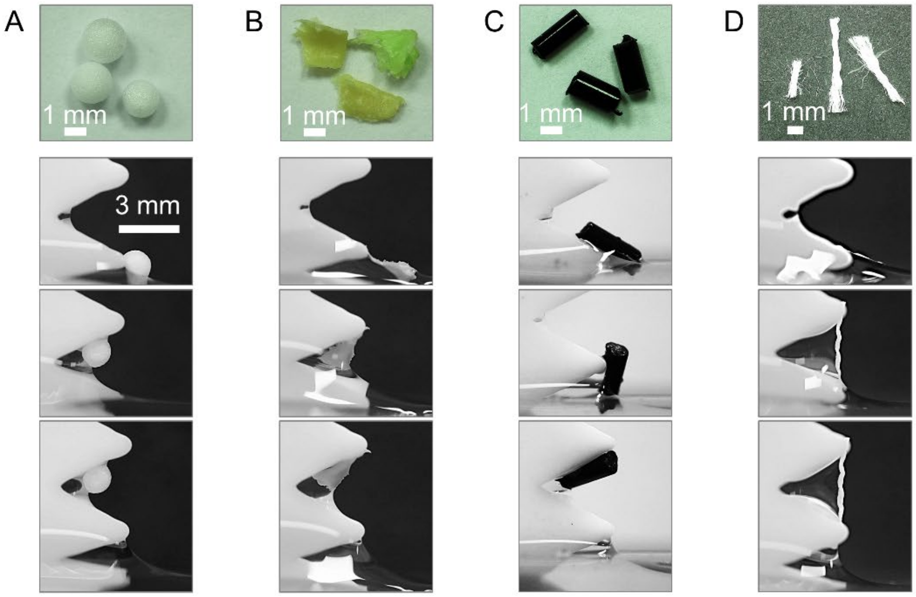

Fig. S10. The side-view of various shapes of MPs being skimmed using a hydrophilic ratchet plate (P = H = 4.5 mm): (**A**) ball-shaped EPS foam, (**B**) irregularly-shaped PE, (**C**) rod-shaped PE, and (**D**) fiber-shaped polyester.

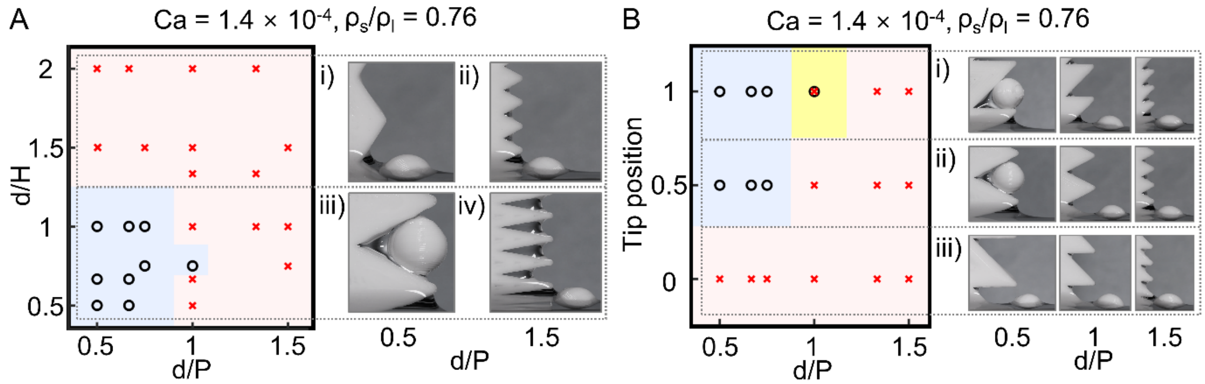

Fig. S11. Skimming capability of various ratchet geometries, including asymmetric designs. (**A**) The regime map of the skimming capability of a ratchet geometry with independent variations in pitch (P) and height (H). (**B**) The skimming capability of an asymmetric ratchet for different asymmetric configurations and normalized ball diameters.

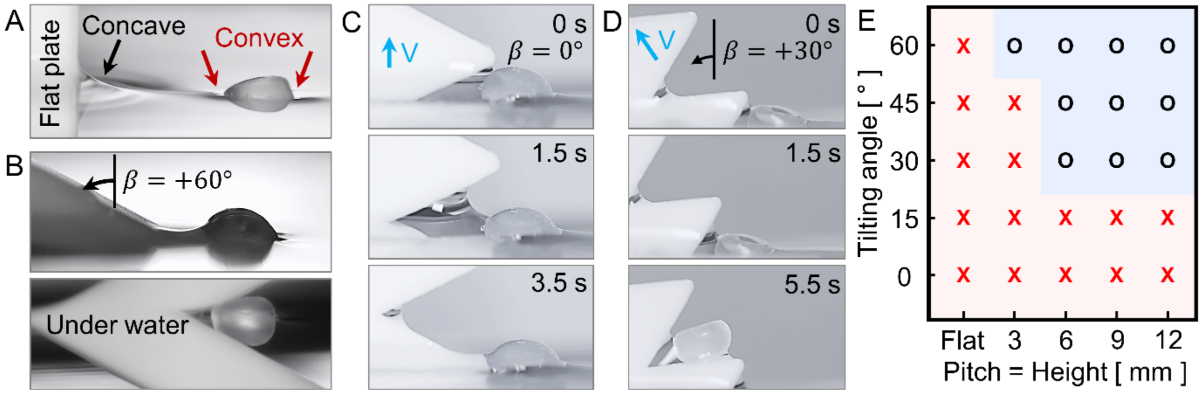

Fig. S12. Enabled skimming of a heavier MP with a convex meniscus by tilting the ratchet. (**A**) When an MP ball of PE pellet forms a convex water meniscus due to its heavier weight, the ball no longer drifts toward the flat plate surface, as the Cheerios effect causes repulsion. An optical image showing the cheerios effect between the flat plate and the MP pellet. (**B**) However, by tilting the plate at a positive angle, the repulsive force caused by the concave water meniscus can be minimized, which allows the MP ball to move freely around the surface of the plate. (**C**) Sequential optical images showing that a 4-mm PE ball (**C**) fails to be skimmed, due to the repulsive Cheerios effect for the vertical lifting (**D**) while succeeded when the ratchet plate is tilted at a positive angle of 30°. (**E**) A graph showing the regime map of skimming capability based on the pitch (P = H) of the ratchet compared to the tilting angle.

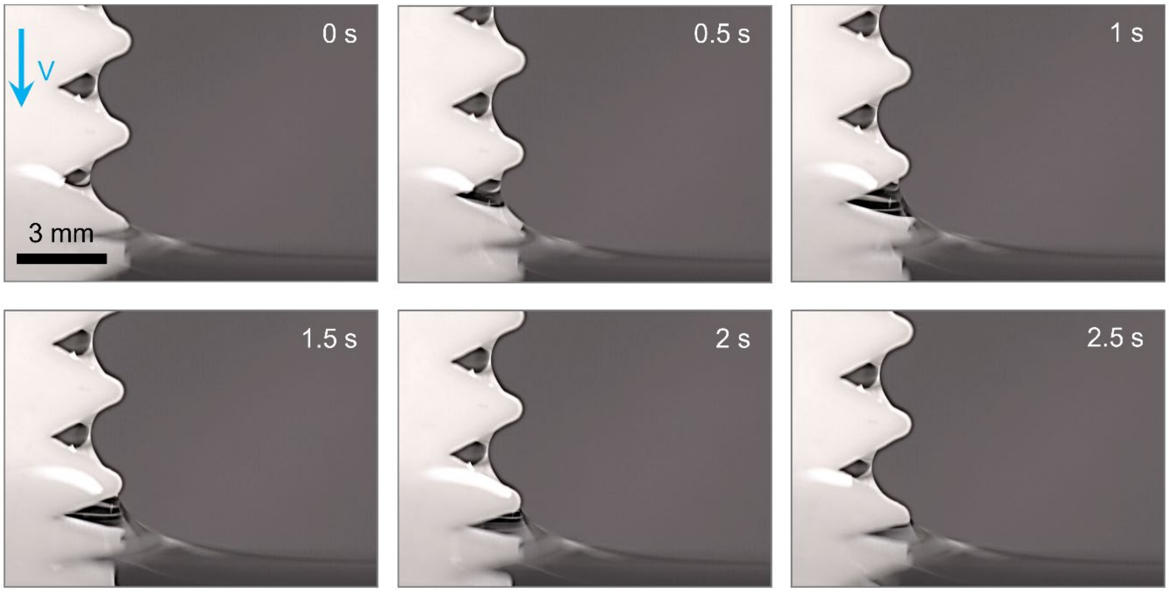

Fig. S13. Optical images of the meniscus evolution sequentially upon dipping the ratchet plate in water in the side view. When the water contained inside the ratchet came into contact with the external water, the meniscus thickened and transformed into an outwardly curved shape with time.

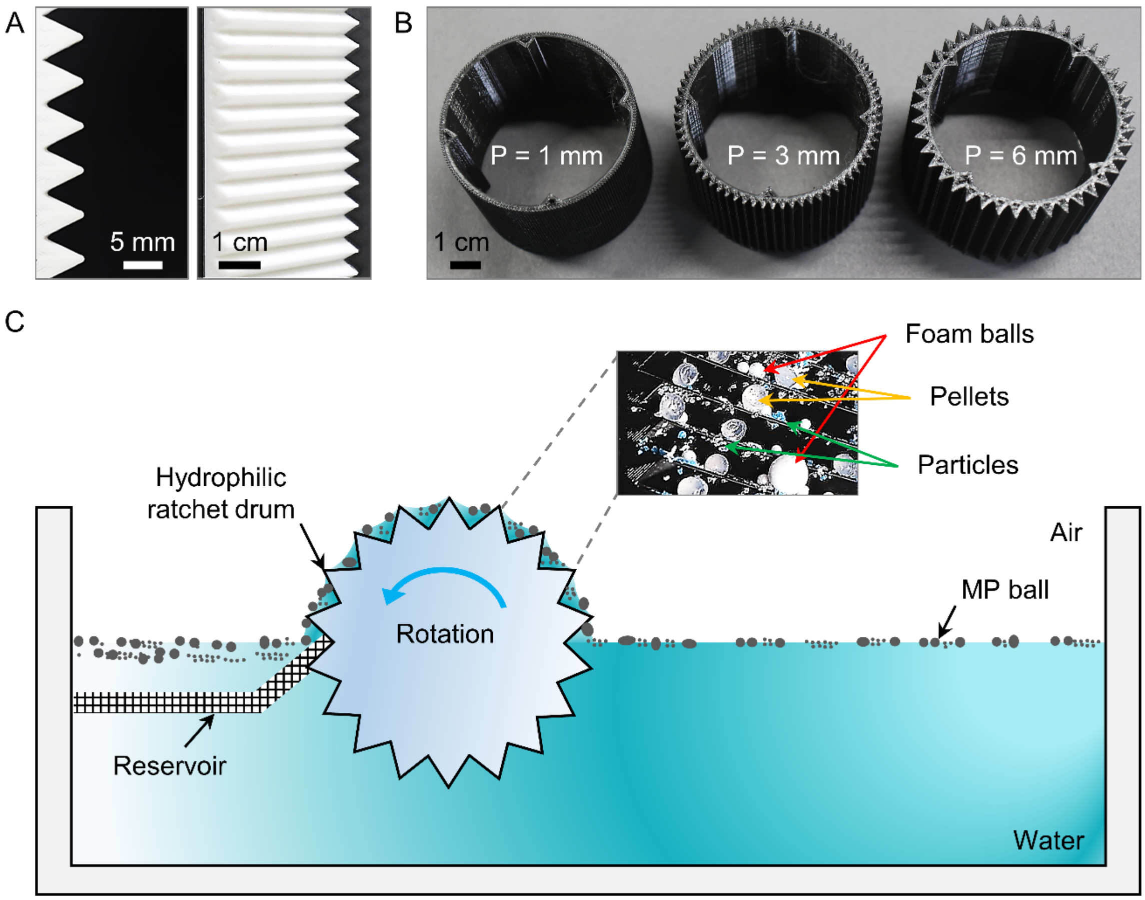

Fig. S14. 3D-printed ratchet structures in various shapes and a setup for direct MP recovery with the ratchet drum system. Optical images showing (**A**) the plate-type ratchet and (**B**) the drum-type ratchet. (**C**) A schematic for the experimental set-up collecting the microplastics by the drum-type ratchet structure. MPs are skimmed from the right side of the ratchet drum and released into the left side, at which the released MPs are collected within the reservoir. An inset image showing the ratchet surface having several types of MP particles such as EPS foam balls, PP pellets, PP particles, PE pellets and PE particles in various size and density (see the data for the density and shape in **Table S2**).

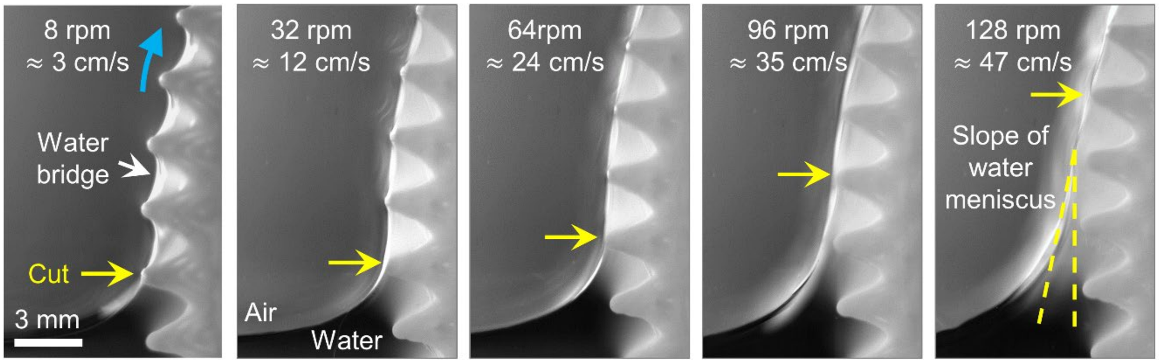

Fig. S15. Effect of the rotation speed on the formation of the capillary bridge. The optical images showing the changes in the shape of the water bridge and water meniscus with increasing rotation speed of the hydrophilic ratchet drum. An arrow in blue indicates the rotation of the drum and arrows in yellow denote the locations where the meniscus cutting occurred.

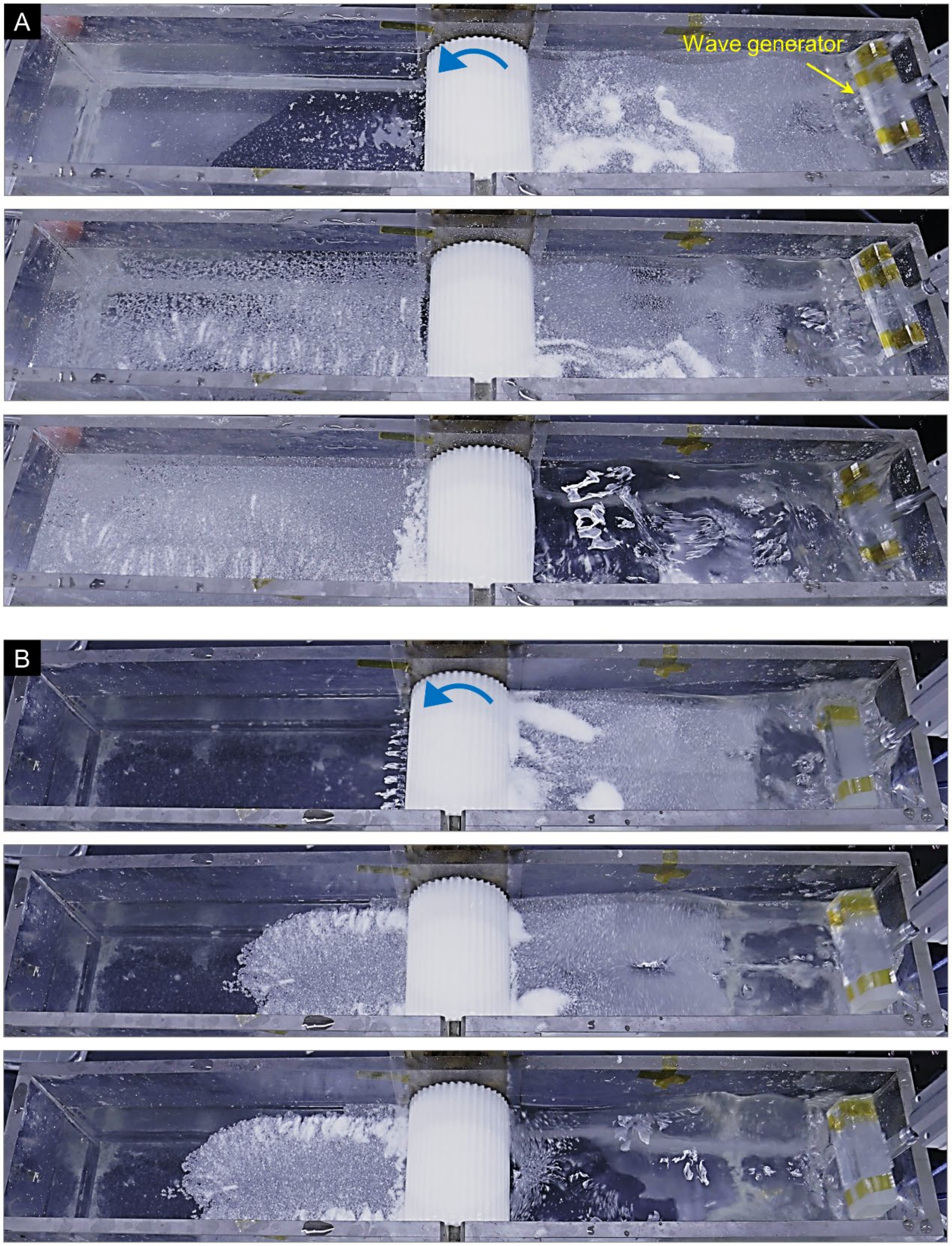

Fig. S16. MP recovery experiments using a hydrophilic ratchet drum with a diameter of 60 mm under wave conditions with wave heights of (A) 10 mm and (B) 26 mm.

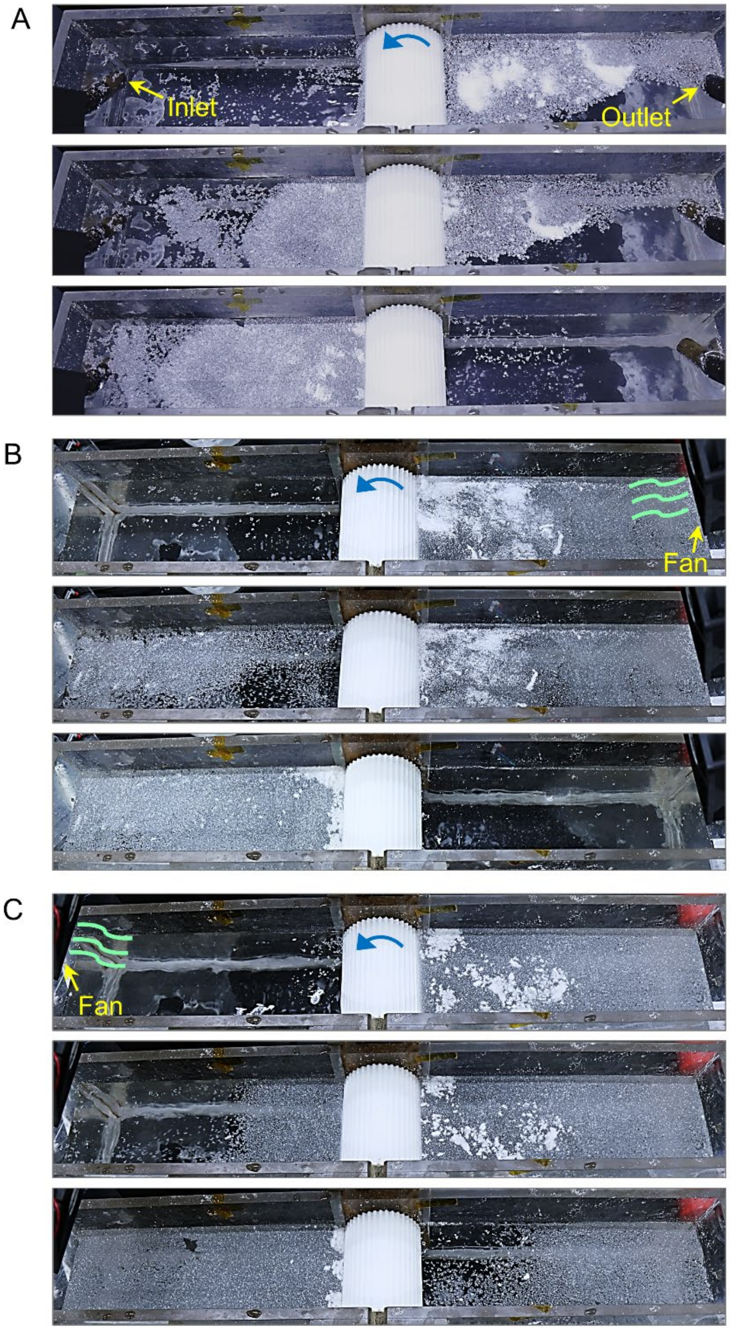

Fig. S17. MP recovery experiment using a hydrophilic ratchet drum. (**A**) Test conducted at a low water temperature of 6 ℃. (**B−C**) Tests with wind applied using a fan at room temperature: (**B**) wind blowing in the direction of skimming and (**C**) wind blowing in the opposite direction.

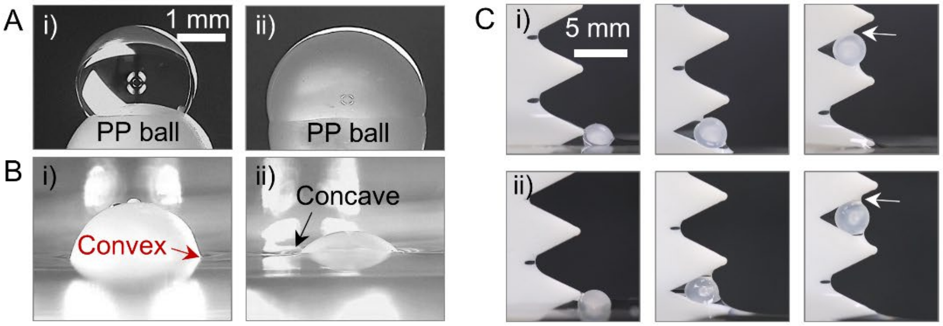

Fig. S18. Skimming of a hydrophilized PP ball. (**A**) Skimming of a hydrophilized i) an untreated pristine PP ball and ii) an oxygen plasma-treated PP ball. (**B**) The changes in the floating state and the shape of surrounding meniscus from convex to concave after the hydrophilization. (**C**) The sequential images of skimming these two different PP balls using a hydrophilic ratchet plate (P = H = 6 mm): i) pristine PP ball and ii) hydrophilized one.

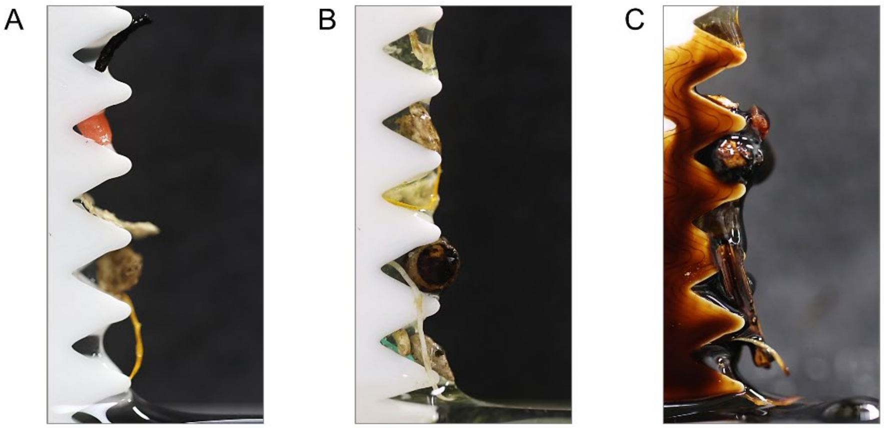

Fig. S19. Skimming MPs using a hydrophilic ratchet plate (P = H = 4.5 mm) with various shapes weathered in three different environments: (**A**) in seawater, (**B**) in seawater with phytoplankton alone, and (**C**) in seawater with a mixture of phytoplankton and a fuel oil of LSFO.

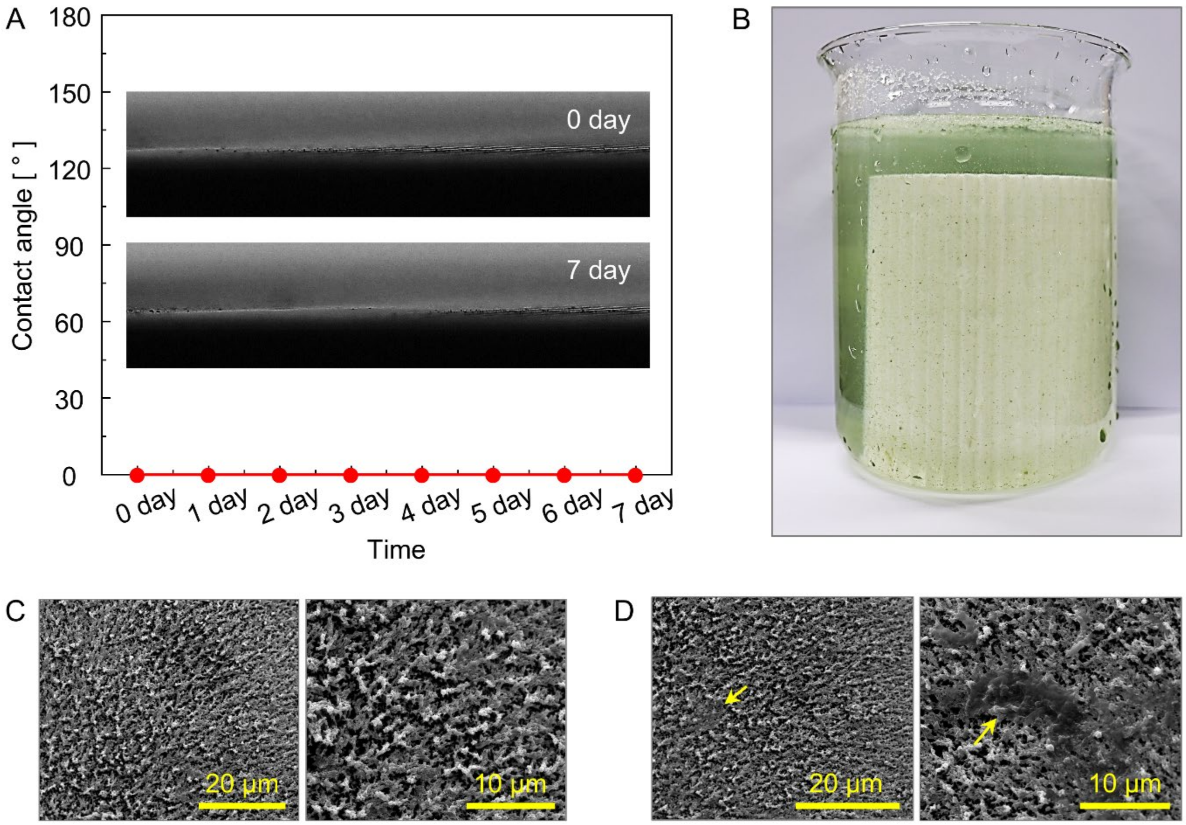

Fig. S20. (**A**) The graph shows that after submerging PLA in seawater and then drying it, the contact angle remained at 0° for up to one week, indicating that its superhydrophilicity was preserved. (**B**) The optical image shows the ratchet drum undergoing weathering for one week in an environment with phytoplankton (Chlorella) to allow for biofilm formation. (**C−D**) The SEM images show the superhydrophilic PLA surface (**C**) before and (**D**) after the 1-week biofouling experiment.

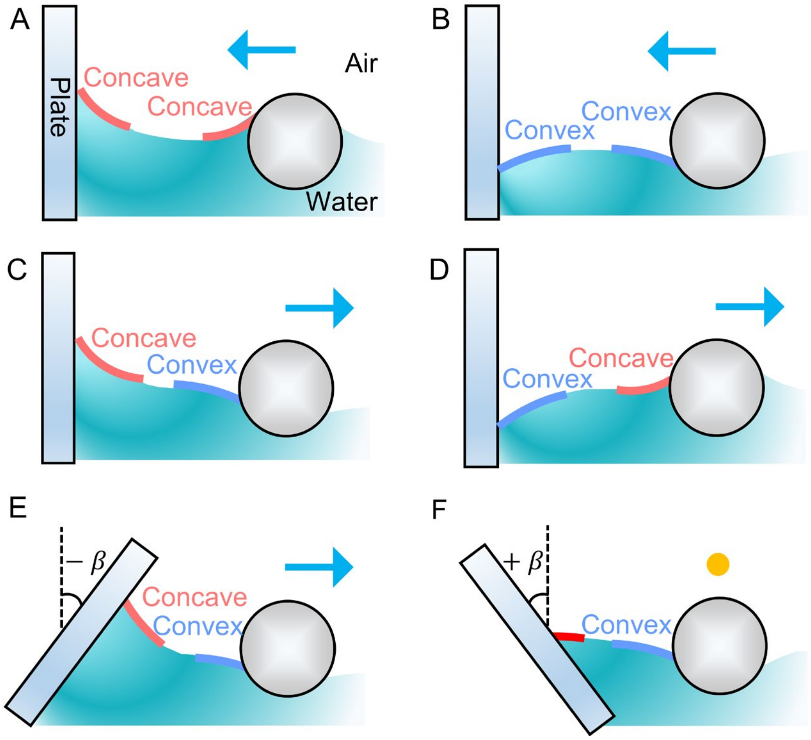

Fig. S21. Schematics of the meniscus formation on the plate and MPs, and the resulting directional force direction. When the menisci between the plate and the ball are formed by combining concave and concave shapes (magenta contour) in (**A**), or convex and convex shapes (blue contour) in (**B**), attractive force is generated. Conversely, when the menisci are formed by combining concave and convex shapes (**C**), or convex and concave shapes (**D**), the plate and the ball experience repulsion force. (**E**) When the plate is tilted in the − direction, the height of the concave water meniscus increases, resulting in an increase in the magnitude of the repulsion force generated. (**F**) When the plate is tilted in the + direction, the height of the concave water meniscus decreases (red contour), resulting in the disappearance of the repulsion force.

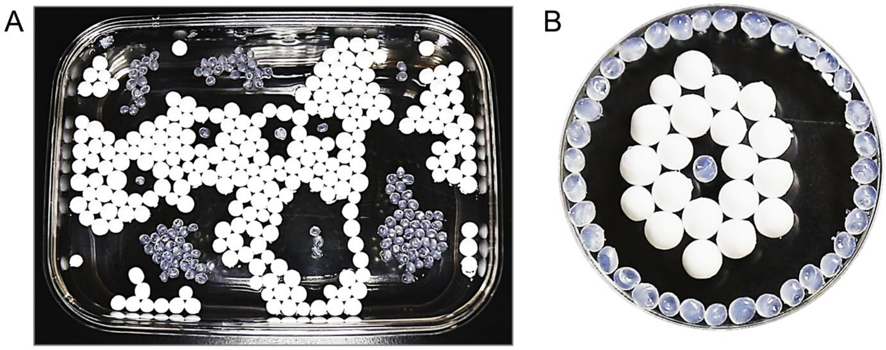

Fig. S22. **Top-down view of an optical image capturing EPS foam balls and PE pellets floating on the water.** Cheerios phenomena with EPS foam balls (white) and PE pellets (translucent) contained in a water-filled hydrophilic glass container (**A**) and a hydrophobic PS container (**B**).

Ball Name	Material	Diameter (mm)	Infill density (%)	Mass (mg)	Density (g cm^{-3})
ED1	EPS foam	2	-	0.15 ± 0.02	0.04 ± 0.005
ED2		6	-	2.6 ± 0.2	0.02 ± 0.005
ED3		10	-	12 ± 0.2	0.02 ± 0.005
ED4		15	-	29 ± 0.5	0.02 ± 0.005
ED5		20	-	73 ± 0.5	0.02 ± 0.005
ED6		30	-	225 ± 0.5	0.02 ± 0.005
PD1I0	Polylactic Acid (3-D printed PLA)	6	0	85 ± 0.5	0.75 ± 0.02
PD1I25			25	88 ± 0.5	0.78 ± 0.02
PD1I50			50	93 ± 0.5	0.82 ± 0.02
PD2I0		9	0	224 ± 0.5	0.59 ± 0.02
PD2I25			25	235 ± 0.5	0.61 ± 0.02
PD2I50			50	299 ± 0.5	0.78 ± 0.02
PD3I0		12	0	400 ± 0.5	0.44 ± 0.02
PD3I25			25	469 ± 0.5	0.52 ± 0.02
PD3I50			50	625 ± 0.5	0.69 ± 0.02

Table. S1. The properties of the ball used in the skimming experiment. The table shows the diameter, mass, and density of 3D printed PLA balls and EPS foam balls. The density of 3D printed PLA balls varied depending on the infill density. ED represents EPS diameter in order, PD represents PLA diameter in order, and I represents infill density.

Plastic	Chemical formula	Density (g cm^{-3})	Contact angle (°)	Shape	
PS	$(C_8H_8)_n$	Foam : 0.02 – 0.04<br>(1.04 – 1.5) (49, 79)	88 – 92 (80)	Foam	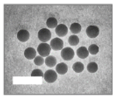
PE	$(C_2H_4)_n$	0.92 – 0.97 (49, 79)	100 – 103(81−83)	Pellet	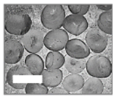
				Particle	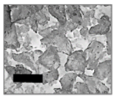
PP	$(C_3H_6)_n$	0.88 – 0.91 (49)	99.8 – 111.5 (84, 85)	Pellet	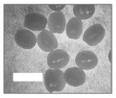
				Particle	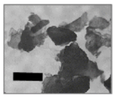

Table. S2. **Material properties of the plastic commonly found floating in the ocean.** The types and conditions of plastics were classified into five categories. The table shows the chemical formula, density, contact angle and shape (*49, 79−85*). The white scale bar is 5 mm, the black scale bar is 0.5 mm.

Classification	Net	Hydrophilic ratchet
Image	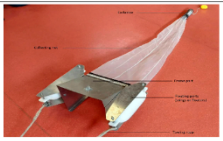	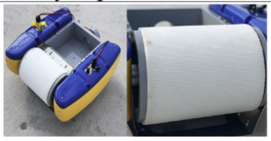
Mechanism	Size-based filtering (*26−28, 65−67, 86−89*)	Capillary skimming
Materials	Nylon, Polyester	Hydrophilic materials such as Polylactic acid (PLA), Polyvinyl alcohol (PVA), cellulose-based materials
Structures	Mesh (1−1000 μm)	Ratchet (milimeter scale, 3−9 mm)
Advantage	Simple operation, Covering open area	Simple operation, Covering open area, Capable of a wide range of sizes (1 μm−5 mm)
Disadvantage	Clogging issue, Pressure issue depending on the mesh size, Long and thin particles such as fibers, may escape depending on their orientation, Risk of sea creatures being caught.	Limited for the MPs floating on the water surface
Cost	350−450 $/m^2$	500−600 $/m^2$
Application	Used for sampling MPs in general rather than cleaning it	Can be used for both cleaning and sampling MPs
How to use	Operated through towing by a ship or drone	Operated through attachment to a drone

Table. S3. Comparative analysis between size-based filtration (widely used for the MP collection) and the hydrophilic ratchet skimming.

Descriptions for supplementary movies

Movie S1. Collecting the various MPs using the hydrophilic ratchet drum. A movie clip shows the recovery of the various MPs floating on water. The ratchet drum recovered EPS foam balls, PP pellets, PP particles, PE pellets, and PE particles (played at 1x speed).

Movie S2. EPS foam ball being skimmed by the hydrophilic ratchet plate. A movie clip shows a series of processes where EPS foam ball was skimmed and released using a ratchet plate. (played at 1x speed).

Movie S3. Skimming small and large EPS foam balls using a hydrophilic flat plate. A movie clip shows the skimming of EPS foam balls with diameters of 2 mm and 5 mm at a lifting speed of 1 mm/s using a hydrophilic flat plate. (played at 1x speed).

Movie S4. Flow vector field generated while lifting the ratchet plate. A movie clip shows the flow vector field generated by the lifting of the ratchet plate at a speed of 50 mm/s (played at 1/75x speed).

Movie S5. Flow vector field generated while lifting the flat plate. A movie clip shows the flow vector field generated by the lifting of the flat plate at a speed of 50 mm/s (played at 1/75x speed).

Movie S6. Capillary water bridge ruptured and skimming failed. A movie clip shows that when the size of the ratchet and the ball became too large, the capillary water bridge inside the ratchet ruptured and skimming failed (played at 1x speed).

Movie S7. The ratchet plate failed to skim a single PE pellet (P = H = 6 mm). A movie clip shows the failure to skim a single PE pellet (played at 1x speed).

Movie S8. Tilting the ratchet plate at + 30° to skim a single PE pellet (P = H = 6 mm). A movie clip shows the tilted ratchet plate skimming a single PE pellet (played at 1x speed).

Movie S9. Skimming and release behavior of PP on the hydrophilic ratchet drum. A movie clip shows the skim and release behavior of the MPs floating on water. It exhibited a skimming behavior for the first 4-5 seconds, followed by a releasing behavior (played at 1x speed).

Movie S10. Marine robot cleaner equipped with a water-bridged ratchet for skimming microplastics. A movie clip demonstrates that the front ratchet drum of the lab-scale prototype effectively skimmed MPs of various sizes ranging from 50 μm to 5 mm while moving forward.

References

1. Carpenter, E. J., Anderson, S. J., Harvey, G. R., Miklas, H. P. & Peck, B. B. Polystyrene spherules in coastal waters. *Science* **178**, 749–750 (1972).
2. Thompson, R. C., Olsen, Y., Mitchell, R. P., Davis, A., Rowland, S. J., John, A. W., McGonigle, D. & Russell, A. E. Lost at sea: where is all the plastic? *Science* **304**, 838–838 (2004).
3. Do Sul, J. A. I. & Costa, M. F. The present and future of microplastic pollution in the marine environment. *Environ. Pollut.* **185**, 352–364 (2014).
4. Perren, W., Wojtasik, A. & Cai, Q. Removal of microbeads from wastewater using electrocoagulation. *ACS omega* **3**, 3357–3364 (2018).
5. Wang, C., Zhao, J. & Xing, B. Environmental source, fate, and toxicity of microplastics. *J. Hazard. Mater.* **407**, 124357 (2021).
6. MacLeod, M., Arp, H. P. H., Tekman, M. B. & Jahnke, A. The global threat from plastic pollution. *Science* **373**, 61–65 (2021).
7. Miller, M. E., Kroon, F. J. & Motti, C. A. Recovering microplastics from marine samples: a review of current practices. *Mar. pollut. bull.* **123**, 6–18 (2017).
8. Andrady, A. L. Microplastics in the marine environment. *Mar. pollut. bull.* **62**, 1596–1605 (2011).
9. Koelmans, A. A., Redondo-Hasselerharm, P. E., Nor, N. H. M., de Ruijter, V. N., Mintenig, S. M. & Kooi, M. Risk assessment of microplastic particles. *Nat. Rev. Mater.* **7**, 138–152 (2022).
10. Gigault, J., El Hadri, H., Nguyen, B., Grassl, B., Rowenczyk, L., Tufenkji, N., Feng, S. & Wiesner, M. Nanoplastics are neither microplastics nor engineered nanoparticles. *Nat. Nanotechnol.* **16**, 501–507 (2021).
11. Turner, A. Foamed polystyrene in the marine environment: sources, additives, transport, behavior, and impacts. *Environ. Sci. Technol.* **54**, 10411–10420 (2020).
12. Vuori, L. & Ollikainen, M. How to remove microplastics in wastewater? A cost-effectiveness analysis. *Ecol. Econ.* **192**, 107246 (2022).
13. Li, C., Busquets, R. & Campos, L. C. Assessment of microplastics in freshwater systems: A review. *Sci. Total Environ.* **707**, 135578 (2020).
14. Leslie, H. A., Van Velzen, M. J., Brandsma, S. H., Vethaak, A. D., Garcia-Vallejo, J. J. & Lamoree, M. H. Discovery and quantification of plastic particle pollution in human blood. *Environ. Int.* **163**, 107199 (2022).
15. Vethaak, A. D. & Legler, J. Microplastics and human health. *Science* **371**, 672–674 (2021).
16. Adelodun, A. A. Plastic recovery and utilization: From ocean pollution to green economy. *Front. environ. sci.* **9**, 683403 (2021).
17. Wang, J., Liu, X., Li, Y., Powell, T., Wang, X., Wang, G. & Zhang, P. Microplastics as contaminants in the soil environment: A mini-review. *Sci. Total Environ.* **691**, 848–857 (2019).
18. Wright, S. L., Thompson, R. C. & Galloway, T. S. The physical impacts of microplastics on marine organisms: a review. *Environ. pollut.* **178**, 483–492 (2013).
19. Peda, C., Caccamo, L., Fossi, M. C., Gai, F., Andaloro, F., Genovese, L., Perdichizzi, A., Romeo, T. & Maricchiolo, G. Intestinal alterations in European sea bass Dicentrarchus labrax (Linnaeus, 1758) exposed to microplastics: preliminary results. *Environ. pollut.* **212**, 251–256 (2016).
20. Nguyen, B., Claveau-Mallet, D., Hernandez, L. M., Xu, E. G., Farner, J. M. & Tufenkji, N. Separation and analysis of microplastics and nanoplastics in complex environmental samples. *Acc. Chem. Res.* **52**, 858–866 (2019).
21. Prata, J. C., Da Costa, J. P., Duarte, A. C. & Rocha-Santos, T. Methods for sampling and detection of microplastics in water and sediment: A critical review. *TrAC, Trends Anal. Chem.* **110**, 150–159 (2019).
22. Kaiser, D., Kowalski, N. & Waniek, J. J. Effects of biofouling on the sinking behavior of microplastics. *Environ. Res. Lett.* **12**, 124003 (2017).
23. Liu, Y., Wang, B., Pileggi, V. & Chang, S. Methods to recover and characterize microplastics in wastewater treatment plants. *Case Stud. Chem. Environ. Eng.* **5**, 100183 (2022).

24. Zhang, W., Zhang, S., Wang, J., Wang, Y., Mu, J., Wang, P., Lin, X. & Ma, D. Microplastic pollution in the surface waters of the Bohai Sea, China. *Environ. pollut.* **231**, 541–548 (2017).
25. Oluwoye, I., Tanaka, S. & Okuda, K. Pilot-scale performance of gravity-driven ultra-high flux fabric membrane systems for removing small-sized microplastics in wastewater treatment plant effluents. *J. Environ. Manage.* **363**, 121438 (2024).
26. Uchida, K., Kuroda, M. & Tokai, T. Comparison of microplastic sampling performance between a neuston net and a manta net. *J. Fish. Eng.* **59**, 19–26 (2022).
27. Montoto-Martínez, T., Meléndez-Díez, C., Melián-Ramírez, A., Hernández-Brito, J. J. & Gelado-Caballero, M. D. Comparison between the traditional Manta net and an innovative device for microplastic sampling in surface marine waters. *Mar. Pollut. Bull.* **185**, 114237 (2022).
28. Pasquier, G., Doyen, P., Kazour, M., Dehaut, A., Diop, M., Duflos, G. & Amara, R. Manta net: The golden method for sampling surface water microplastics in aquatic environments. *Front. environ. sci.* **10**, 811112 (2022).
29. Wang, T., Joo, H.-J., Song, S., Hu, W., Keplinger, C. & Sitti, M. A versatile jellyfish-like robotic platform for effective underwater propulsion and manipulation. *Sci. Adv.* **9**, eadg0292 (2023).
30. Bhardwaj, N. & Bhaskarwar, A. N. A review on sorbent devices for oil-spill control. *Environ. pollut.* **243**, 1758–1771 (2018).
31. Hoang, A. T., Nguyen, X. P., Duong, X. Q. & Huynh, T. T. Sorbent-based devices for the removal of spilled oil from water: a review. *Environ. Sci. Pollut. Res.* **28**, 28876–28910 (2021).
32. Ko, T.-J., Cho, S., Kim, S. J., Lee, Y. A., Jo, W., Kim, H.-Y., Yang, S., Oh, K. H. & Moon, M.-W. Direct recovery of spilled oil using hierarchically porous oil scoop with capillary-induced anti-oil-fouling. *J. Hazard. Mater.* **410**, 124549 (2021).
33. Lee, Y. A., Park, Y. C., Kwon, O., Kim, S. J., Chung, S. & Moon, M.-W. Hygroscopic ramie fabrics for recovering highly viscous low sulfur fuel oil. *Environ. Pollut.* **308**, 119668 (2022).
34. Lee, Y. A., Cho, S., Choi, S., Kwon, O.-C., Yoon, S. M., Kim, S. J., Park, K.-C., Chung, S. & Moon, M.-W. Slippery, Water-Infused Membrane with Grooved Nanotrichomes for Lubricating-Induced Oil Repellency. *Adv. Sci.* **9**, 2103950 (2022).
35. Vella, D. & Mahadevan, L. The "cheerios effect" *Am. J. Phys.* **73**, 817–825 (2005).
36. Ho, I., Pucci, G. & Harris, D. M. Direct measurement of capillary attraction between floating disks. *Phys. Rev. Lett.* **123**, 254502 (2019).
37. Meseguer, J., Slobozhanin, L. & Perales, J. A review on the stability of liquid bridges. *Adv. Space Res.* **16**, 5–14 (1995).
38. Park, J., Lee, C., Lee, S., Cho, H., Moon, M.-W. & Kim, S. J. Clogged water bridges for fog harvesting. *Soft Matter* **17**, 136–144 (2021).
39. Broesch, D. J. & Frechette, J. From concave to convex: capillary bridges in slit pore geometry. *Langmuir* **28**, 15548–15554 (2012).
40. Reddy, S., Schunk, P. R. & Bonnecaze, R. T. Dynamics of low capillary number interfaces moving through sharp features. *Phys. Fluids* **17** (2005).
41. Seebergh, J. E. & Berg, J. C. Dynamic wetting in the low capillary number regime. *Chem. Eng. Sci.* **47**, 4455–4464 (1992).
42. Chatzis, I. & Morrow, N. R. Correlation of capillary number relationships for sandstone. *Soc. Pet. Eng. J.* **24**, 555–562 (1984).
43. Schnurr, R. E., Alboiu, V., Chaudhary, M., Corbett, R. A., Quanz, M. E., Sankar, K., Srain, H. S., Thavarajah, V., Xanthos, D. & Walker, T. R. Reducing marine pollution from single-use plastics (SUPs): A review. *Mar. Pollut. Bull.* **137**, 157–171 (2018).
44. Xanthos, D. & Walker, T. R. International policies to reduce plastic marine pollution from single-use plastics (plastic bags and microbeads): A review. *Mar. Pollut. Bull.* **118**, 17–26 (2017).
45. Lebreton, L., Slat, B., Ferrari, F., Sainte-Rose, B., Aitken, J., Marthouse, R., Hajbane, S., Cunsolo, S., Schwarz, A., Levivier, A., et al. Evidence that the Great Pacific Garbage Patch is rapidly accumulating plastic. *Sci. Rep.* **8**, 1–15 (2018).

46. Almroth, B. C. & Eggert, H. Marine plastic pollution: sources, impacts, and policy issues. *Rev. Environ. Econ. Policy.* **13,** 317-326 (2019).
47. Karlsson, T. M., Arneborg, L., Broström, G., Almroth, B. C., Gipperth, L. & Hassellöv, M. The unaccountability case of plastic pellet pollution. *Mar. Pollut. Bull.* **129**, 52–60 (2018).
48. Sewwandi, M., Hettithanthri, O., Egodage, S., Amarathunga, A. & Vithanage, M. Unprecedented marine microplastic contamination from the X-Press Pearl container vessel disaster. *Sci. Total Environ.* **828**, 154374 (2022).
49. Stubbins, A., Law, K. L., Mu˜noz, S. E., Bianchi, T. S. & Zhu, L. Plastics in the Earth system. *Science* **373**, 51–55 (2021).
50. Butt, H. J. & Kappl, M. Normal capillary forces. *Adv. Colloid Interface Sci.* **146**, 48-60 (2009).
51. Mielniczuk, B., Hueckel, T. & El Youssoufi, M. S. Laplace pressure evolution and four instabilities in evaporating two-grain liquid bridges. *Powder Technol.* **283**, 137-151 (2015).
52. Mishima, S., Iikura, H. & Ougizawa, T. Study of adhesion between microspheres and rubber surfaces accompanied by meniscus formation and sedimentation. *Appl. Adhes. Sci.* **5**, 5 (2017).
53. Atugoda, T., Vithanage, M., Wijesekara, H., Bolan, N., Sarmah, A. K., Bank, M. S., You, S. & Ok, Y. S. Interactions between microplastics, pharmaceuticals and personal care products: Implications for vector transport. *Environ. Int.* **149**, 106367 (2021).
54. Hou, Y., Xue, B., Guan, S., Feng, S., Geng, Z., Sui, X., Lu, J., Gao, L. & Jiang, L. Temperature-controlled directional spreading of water on a surface with high hysteresis. *NPG Asia Mater.* **5**, e77 (2013).
55. Ouenzerfi, S. & Harmand, S. Experimental Droplet Study of Inverted Marangoni Effect of a Binary Liquid Mixture on a Nonuniform Heated Substrate. *Langmuir* **32**, 2378-2388 (2016).
56. Tu, C., Chen, T., Zhou, Q., Liu, Y., Wei, J., Waniek, J. J. & Luo, Y. Biofilm formation and its influences on the properties of microplastics as affected by exposure time and depth in the seawater. *Sci. Total Environ.* **734**, 139237 (2020).
57. Van Melkebeke, M., Janssen, C. & De Meester, S. Characteristics and sinking behavior of typical microplastics including the potential effect of biofouling: implications for remediation. *Environ. Sci. Technol.* **54**, 8668–8680 (2020).
58. Pete, A. J., Brahana, P. J., Bello, M., Benton, M. G. & Bharti, B. Biofilm formation influences the wettability and settling of microplastics. *Environ. Sci. Technol. Lett.* **10**, 159–164 (2022).
59. Hossain, M. R., Jiang, M., Wei, Q. & Leff, L. G. Microplastic surface properties affect bacterial colonization in freshwater. *J. Basic Microbiol.* **59**, 54–61 (2019).
60. Vedachalam, S., Baquerizo, N. & Dalai, A. K. Review on impacts of low sulfur regulations on marine fuels and compliance options. *Fuel* **310**, 122243 (2022).
61. Zhang, Y., Jiang, H., Bian, K., Wang, H. & Wang, C. Is froth flotation a potential scheme for microplastics removal? Analysis on flotation kinetics and surface characteristics. *Sci. Total Environ.* **792**, 148345 (2021).
62. Jiang, H., Zhang, Y., Bian, K., Wang, C., Xie, X., Wang, H. & Zhao, H. Is it possible to efficiently and sustainably remove microplastics from sediments using froth flotation? *Chem. Eng. J.* **448**, 137692 (2022).
63. Pandey, A., Chen, Z.-Y., Yuk, J., Sun, Y., Roh, C., Takagi, D., Lee, S. & Jung, S. Optimal free-surface pumping by an undulating carpet. *Nat. Commun*. **14**, 7735 (2023).
64. Punzmann, H., Francois, N., Xia, H., Falkovich, G. & Shats, M. Generation and reversal of surface flows by propagating waves. *Nat. Phys.* **10**, 658–663 (2014).
65. Imbulana, S., Tanaka, S., Moriya, A. & Oluwoye, I. Inter-event and intra-event dynamics of mi-croplastic emissions in an urban river during rainfall episodes. *Environ. Res.* **243**, 117882 (2024).
66. Pasquier, G., Doyen, P., Carlesi, N. & Amara, R. An innovative approach for microplastic sampling in all surface water bodies using an aquatic drone. *Heliyon* **8** e11662 (2022).
67. Brown, D. & Cheng, L. New net for sampling the ocean surfa3ce. *Mar. Ecol. Prog. Ser.* **5**, 225–227 (1981).

68. Du, R., Sun, X., Lin, H. & Pan, Z. Assessment of manta trawling and two newly-developed surface water microplastic monitoring techniques in the open sea. *Sci. Total Environ.* **842**, 156803 (2022).
69. Thielicke, W. & Sonntag, R. Particle Image Velocimetry for MATLAB: Accuracy and enhanced algorithms in PIVlab. *J. Open Res. Softw.* **9**, 9-12 (2021).
70. Zhao, C., Cai, L., Nie, M., Shang, L., Wang, Y., Zhao, Y. Cheerios Effect Inspired Microbubbles as Suspended and Adhered Oral Delivery Systems. *Adv. Sci.* **8**, 2004184 (2021).
71. Xie, G., Li, P., Kim, P. Y., Gu, P. Y., Helms, B. A., Ashby, P. D., Jiang, L. & Russell, T. P. Continuous, autonomous subsurface cargo shuttling by nature-inspired meniscus-climbing systems. *Nat. Chem.* **14**, 208-215 (2022).
72. Liu, J. & Li, S., Capillarity-driven migration of small objects: A critical review. *European Physical Journal E* **42**, 1 (2019).
73. Hu, D. L. & Bush, J. W. M. Meniscus-climbing insects. *Nature* **437**, 733-736 (2005).
74. Peruzzo, P., Defina, A. & Nepf, H. Capillary trapping of buoyant particles within regions of emergent vegetation. *Water Resour. Res.* **48**, W07512 (2012).
75. Liu, X., Zeng, Y. & Huai, W. Floating seed dispersal in open channel flow with emergent vegetation. *Ecohydrology* **12**, e2038 (2019).
76. Katoh, K., Fujita, H. & Imazu, E. Motion of a particle floating on a liquid meniscus surface. *J. Fluids Eng. Trans. ASME.* **114**, 411 (1992).
77. Kralchevsky, P. & Nagayama, K. Particles at fluid interfaces and membranes: attachment of colloid particles and proteins to interfaces and formation of two-dimensional arrays. *Elsevier* (2001).
78. Yuan, J., Feng, J. & Cho, S. K. Dielectrowetting control of capillary force (Cheerios effect) between floating objects and wall for dielectric fluid. *Micromachines* **12**, 341 (2021).
79. Li, J. & Favis, B. D. Characterizing co-continuous high density polyethylene/polystyrene blends. *Polymer* **42**, 5047-5053 (2001).
80. Kwok, D. Y., Lam, C. N. C, Li, A., Zhu, K., Wu, R. & Neumann, A. W. Low-rate dynamic contact angles on polystyrene and the determination of solid surface tensions. *Polym. Eng. Sci.* **38**, 1675-1684 (1998).
81. De Geyter, N., Morent, R. & Leys, C. Surface characterization of plasma-modified polyethylene by contact angle experiments and ATR-FTIR spectroscopy. *Surf. Interface Anal.* **40**, 608-611 (2008).
82. Holmes-Farley, S. R., Bain, C. D. & Whitesides, G. M. Wetting of functionalized polyethylene film having ionizable organic acids and bases at the polymer-water interface: Relations between functional group polarity, extent of ionization, and contact angle with water. *Langmuir* **4**, 921-937 (1988).
83. Zdziennicka, A., Szymczyk, K., Krawczyk, J. & Jańczuk B. Some remarks on the solid surface tension determination from contact angle measurements. *Appl. Surf. Sci.* **405**, 88-101 (2017).
84. Schonherr, H., Hruska, Z. & Vancso, G. J. Surface characterization of oxyfluorinated isotactic polypropylene films: Scanning force microscopy with chemically modified probes and contact angle measurements. *Macromolecules* **31**, 3679-3685 (1998).
85. Kwon, O. J., Tang, S., Myung, S. W., Lu, N. & Choi, H. S. Surface characteristics of polypropylene film treated by an atmospheric pressure plasma. *Surf. Coat. Technol.* **192**, 1-10 (2005).
86. Beljanski, A., Cole, C., Fuxa, F., Setiawan, E. & Singh, H. Efficiency and effectiveness of a low-cost, self-cleaning microplastic filtering system for wastewater treatment plants in NCUR Proceedings. 30th National Conference on Undergraduate Research (NCUR), 1388–1395 (2016).
87. Cai, H., Chen, M., Chen, Q., Du, F., Liu, J. & Shi, H. Microplastic quantification affected by structure and pore size of filters. *Chemosphere* **257**, 127198 (2020).

88. Bannick, C. G., Szewzyk, R., Ricking, M., Schniegler, S., Obermaier, N., Barthel, A. K., Altmann, K., Eisentraut, P. & Braun, U. Development and testing of a fractionated filtration for sampling of microplastics in water. *Water Res.* **149**, 650–658 (2019).
89. Wang, Z., Sedighi, M. & Lea-Langton, A. Filtration of microplastic spheres by biochar: removal efficiency and immobilisation mechanisms. *Water Res.* **184**, 116165 (2020).
